# Estimated Dietary Intake of Radionuclides and Health Risks for the Citizens of Fukushima City, Tokyo, and Osaka after the 2011 Nuclear Accident

**DOI:** 10.1371/journal.pone.0112791

**Published:** 2014-11-12

**Authors:** Michio Murakami, Taikan Oki

**Affiliations:** 1 Institute of Industrial Science, The University of Tokyo, Meguro, Tokyo, Japan; 2 Japan Science and Technology Agency, Core Research for Evolutionary Science and Technology (CREST), Chiyoda, Tokyo, Japan; Van Andel Institute, United States of America

## Abstract

The radionuclides released from the Fukushima Daiichi nuclear power plant in 2011 pose a health risk. In this study, we estimated the 1st-year average doses resulting from the intake of iodine 131 (^131^I) and cesium 134 and 137 (^134^Cs and ^137^Cs) in drinking water and food ingested by citizens of Fukushima City (∼50 km from the nuclear power plant; outside the evacuation zone), Tokyo (∼230 km), and Osaka (∼580 km) after the accident. For citizens in Fukushima City, we considered two scenarios: Case 1, citizens consumed vegetables bought from markets; Case 2, citizens consumed vegetables grown locally (conservative scenario). The estimated effective doses of ^134^Cs and ^137^Cs agreed well with those estimated through market basket and food-duplicate surveys. The average thyroid equivalent doses due to ingestion of ^131^I for adults were 840 µSv (Case 1) and 2700 µSv (Case 2) in Fukushima City, 370 µSv in Tokyo, and 16 µSv in Osaka. The average effective doses due to ^134^Cs and ^137^Cs were 19, 120, 6.1, and 1.9 µSv, respectively. The doses estimated in this study were much lower than values reported by the World Health Organization and the United Nations Scientific Committee on the Effects of Atomic Radiation, whose assessments lacked validation and full consideration of regional trade in foods, highlighting the importance of including regional trade. The 95th percentile effective doses were 2–3 times the average values. Lifetime attributable risks (LARs) of thyroid cancers due to ingestion were 2.3–39×10^−6^ (Case 1) and 10–98×10^−6^ (Case 2) in Fukushima City, 0.95–14×10^−6^ in Tokyo, and 0.11–1.3×10^−6^ in Osaka. The contributions of LARs of thyroid cancers due to ingestion were 7.5%–12% of all exposure (Case 1) and 12%–30% (Case 2) in Fukushima City.

## Introduction

Radionuclides were released from the Tokyo Electric Power Company's Fukushima Daiichi nuclear power plant, mainly on 15 March 2011, after the Great East Japan Earthquake and tsunami on 11 March. They were diffused into the atmosphere, deposited mainly through precipitation, and incorporated into surface waters, drinking waters, agricultural crops, and aquatic organisms. Radionuclides have been detected in drinking water and foods in Fukushima and other prefectures in Japan, including Tokyo [1], [2], [3]. Radioactive iodine 131 (^131^I), which has a short half-life (8.04 d [4]), can contaminate drinking water and foods immediately after its release, whereas radioactive cesium 134 and 137 (^134^Cs and ^137^Cs), which have longer half-lives (2.06 and 30 y, respectively [4]), cause contamination over longer periods of time. Although other radionuclides such as strontium 90 (^90^Sr), ruthenium 106 (^106^Ru), and plutonium have half-lives of >1 y, in the year after the accident ^134^Cs and ^137^Cs contributed an estimated 84% to 88% of the total radiation dose from radionuclides with half-lives of >1 y in the diet [5].

In response to the accident, the Japanese government announced provisional “indices relating to limits on food and drink ingestion” on 17 March 2011 [1]. The government began releasing monitoring data on radionuclide concentrations in foods on 19 March and restricted the distribution of foods collected in some municipalities from 21 March, as the foods exceeded the limits. Some municipalities in Fukushima prefecture voluntarily imposed more stringent limits. In addition, some local governments, including the Tokyo Metropolitan Government, distributed bottled water to protect the health of citizens, particularly infants, against the high concentrations of ^131^I detected in drinking water.

Among the exposure pathways, internal exposure from dietary intake has not been fully elucidated. External exposure can be determined from personal dosimeters [6], calculated from ambient dose monitoring and shielding factors [7], or estimated by using numerical dispersion models [8]. Internal exposure from inhalation can be estimated by air monitoring [9] or by numerical dispersion models [8], [10]. Internal exposure to Cs can be measured by whole-body-counter surveys; however, the Cs burdens in most inhabitants of Fukushima have been below the detection limit [11], [12]. Internal exposure from the intake of water and food can be determined from market basket surveys (i.e., foods are purchased from the market according to consumption patterns) or food-duplicate surveys (i.e., cooked foods are collected from home) [1], [13], but exposure immediately after the accident could not be evaluated by these methods because of the lack of samples. On the other hand, individual foods were monitored just after the accident for restriction of the distribution of foods, as described above. On the basis of the monitoring data, working groups of the Ministry of Health, Labour and Welfare [14] and the World Health Organization (WHO) [15] approximated the nationwide average internal exposure to radionuclides from foods. The United Nations Scientific Committee on the Effects of Atomic Radiation (UNSCEAR) also recently reported that an increased risk of thyroid cancer for infants and children in particular is of concern, that most of the absorbed dose to the thyroid was received during the first year after the accident, and that the major route of exposure was ingestion; e.g., absorbed dose to thyroid in the first year for 1-year-olds in Fukushima Prefecture (districts not evacuated): external + inhalation, 0.2–19 mGy; ingestion, 33 mGy [16]. However, the regional trade in foods was not fully considered in these assessments despite the low food self-sufficiency rates in Japan (54% for major grains, 39% on basis of total energy [17]), and no validations based on the results of market basket, food-duplicate, or whole-body-counter surveys were done. More detailed estimates and validation are required on account of regional variations in concentrations in drinking water and foods, the regional trade in foods, and variations in individual exposure. Furthermore, recent screening of all children in Fukushima Prefecture identified thyroid cancer in 90 of more than 280 000 children (an incidence of 313 per 1 million) by March 2014 [18]. Although this incidence might be attributable to increased screening by advanced ultrasound techniques [18], an increase incidence of cancer in future, especially thyroid cancer in children, is a matter of concern. The the contribution of cancer risk from ingestion needs to be understood.

To provide more detail, we estimated the 1st-year average doses due to intake of drinking water and foods after the accident from radionuclide concentrations measured in water and foods and the effects of countermeasures (i.e., restriction of food distribution, voluntary withholding of rice, and distribution of bottled water for infants). We previously reported the average thyroid doses resulting from the intake of ^131^I by citizens of Tokyo and the effects of countermeasures [19]. Here, we estimated the intake of ^131^I, ^134^Cs, and ^137^Cs by the citizens of Fukushima City, Tokyo, and Osaka, which differ in the regional trade in foods. The results were validated against observations in market basket, food-duplicate, and whole-body-counter surveys. By comparing the results with reports by WHO and UNSCEAR, whose assessments lacked validation and full consideration of regional trade in foods, we evaluated the role of regional trade in foods. We also estimated variations in intakes by using a Monte Carlo simulation. Finally, we assessed the cancer risks due to intake. We considered all sources of exposure for citizens in Fukushima City, and evaluated the contributions of cancer risks due to ingestion to the overall risk.This information will help to address current concerns of citizens and be useful for establishing the regulation of foods and drinking water.

## Methods

### Intake pathway

To assess intake via the ingestion of drinking water and foods, we estimated the thyroid equivalent dose and effective dose of ^131^I and the effective dose of ^134^Cs and ^137^Cs among citizens of Fukushima City (∼50 km from the nuclear power plant; capital of prefecture), Tokyo (∼230 km), and Osaka (∼580 km). All three cities, including Fukushima City, were outside the evacuation zone. Citizens were classified into ten groups based on age, sex, and pregnancy: <1-y-old infants, males and females aged 1–6, 7–12, 13–18, and ≥19 y old, and pregnant females.

Monitoring data on drinking water and foods were obtained from ref [1], [2], [3], [20]. Drinking water and foods were classified into 18 categories [21]: drinking water, rice, other grains, potato, leafy vegetables, root crops, beans, fruit vegetables, milk, dairy products, formula milk, beef, pork, chicken, chicken eggs, fresh fisheries products, marine products, and others. “Fruit vegetables” included vegetable-like fruits such as tomatoes and fruits such as persimmons and apples. They were then further classified into sub-categories based on governmental restrictions [1]: leafy vegetables were divided into “spinach”, “garland chrysanthemum and ging-geng-cai”, “mustard spinach and non-heading lettuce”, “heading leafy vegetables”, “broccoli and cauliflower”, and “naganegi onion, chive, and asparagus”; root crops into “turnip”, “bamboo shoots”, and “other root crops”; fruit vegetables into “kiwifruit”, “chestnut”, and “other fruit vegetables”; fresh fisheries products into “wild *ayu*, wild Japanese dace, and wild landlocked *masu* salmon” and “other fresh fisheries products”; and other into “tea”, “shiitake mushroom (virgin wood)”, and “other mushrooms”.

### Drinking water

The average doses (thyroid equivalent dose of ^131^I and the effective dose of ^131^I, ^134^Cs, and ^137^Cs) from drinking water were estimated as: 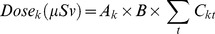
(1)where *k* is the radionuclide, *A_k_* is the dose coefficient for radionuclide *k* (µSv/Bq) (Table S1), *B* is the daily consumption of drinking water per person (g/d) (Table S2), *t* is the number of days after the accident (consumption date), and *C_kt_* is the concentration of radionuclide *k* in drinking water at *t* days after the accident (Bq/g).

So as not to underestimate the dose, we set *A_k_* for ^131^I on the assumption that the fractional thyroid uptake from blood is 0.3, as used in the determination of control index levels by the Nuclear Safety Commission of Japan [22]. All the dose coefficients are reference values taken from the publications of the International Commission on Radiological Protection (ICRP) (Table S1).

*B* was set at 710 g/d for <1 y, 1000 g/d for 1–6 y, and 1650 g/d for >6 y [22]. The average daily consumption of water in soup and rice was then added [19]: The consumption of water in soup was calculated from the consumption of soup by adults [23] and the ratio of miso (soybean paste) consumption by each age group to that by adults [24]. The consumption of water in rice was calculated from the consumption of rice [21] and the ratio of cooking water to rice [25].

The radionuclide concentrations in drinking water were the values monitored in tap water in Fukushima City [3]; Shinjuku, Tokyo [2]; and Osaka (not detected) [20]. As details of ^134^Cs and ^137^Cs concentrations in tap water in Fukushima City were not available, we estimated them from the ^131^I concentrations and the ratios of ^134^Cs and ^137^Cs to ^131^I in tap water in Tokyo between 19 March and 8 April. Intake was estimated from the date when radionuclides were first detected (16 March in Fukushima City, 18 March in Tokyo). As they were not detected in tap water after 4 May (except on 2 July in Tokyo), intake after then was considered to be negligible.

### Foods

The average doses from foods were estimated by the following equation [19], which is modified to include ^134^Cs and ^137^Cs, product origins, and food categories: 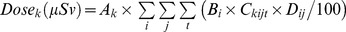
(2)where *k* is the radionuclide, *A_k_* is the dose coefficient for radionuclide *k* (µSv/Bq) (Table S1), *i* is the individual food category, *j* is the individual area (source prefecture or prefectural sub-group), *t* is the number of days after the accident (consumption date), *B_i_* is the daily consumption of food *i* per person (g/d) (Table S2), *C_kijt_* is the arithmetic mean concentration of radionuclide *k* in food *i* in area *j* at *t* days after the accident (Bq/g), and *D_ij_* is the arrival share in an area (the fraction of food *i* in the market that comes from prefecture or area *j*) (%).

#### Daily consumption

For the daily consumption of each food recently reported detailed values were adopted [21]. The daily consumption of the sub-categorized foods was estimated from the daily consumption of the foods in the main categories and the ratio of the amount of each sub-category arriving at the Tokyo Metropolitan Central Wholesale Market to the total in 2010 [26]. The national fishery yield was used to apportion the daily consumption of fresh fisheries products [27]. The daily consumption of mushrooms followed reported values [24]. The daily consumption of tea by ≥19-y-old females was 439 g/d [28], and that by other groups was calculated from that and the ratio of the daily consumption of drinking water excluding water in soup and rice in each group to that in ≥19-y-old females. We assumed that 10 g of leaf is used to make 300 g of tea and that 60% of radionuclides in the leaf enter the tea [14]. The ratio of virgin wood to other substrates used to grow shiitake was used to estimate the daily consumption of “shiitake mushroom (virgin wood)” [29].

#### Radionuclide concentrations

The radionuclide concentrations in food were drawn from >130 000 food monitoring data [1] and rice monitoring data [3]. Details of the number of samples analyzed each month and in each prefecture are given in the Supporting Information and Tables S3 and S4. Foods were washed with water and the edible parts were then analyzed for radionuclides by Ge, NaI or CsI detectors. We considered the intake of radionuclides via the consumption of foods collected in 16 prefectures: Hokkaido, Aomori, Iwate, Miyagi, Akita, Yamagata, Fukushima, Ibaraki, Tochigi, Gunma, Saitama, Chiba, Kanagawa, Tokyo (Metropolis), Yamanashi, and Shizuoka (Figure S1). Analysis of the variations in radionuclide concentrations among foods in Fukushima Prefecture showed that spinach, broccoli and cauliflower, and marine products were heavily contaminated (Figures S2 and S3). Analysis of the regional differences in radionuclide concentrations in spinach showed that foods collected in Fukushima Prefecture and in prefectures in the Kanto region (Ibaraki, Tochigi, Gunma, Saitama, Chiba, Kanagawa, and Tokyo) had much higher levels than those in the other 8 prefectures (Figure S4). The amounts of radionuclides originating from other prefectures or from overseas were regarded as negligible. When radionuclides were not detected in a food, the contribution of radionuclides from that food to the dose was considered to be negligible. The concentrations in each food were classified by source prefecture and date. The prefectures where the distribution of the food was restricted (for example, rice in Fukushima Prefecture) were further classified into prefectural sub-groups. The arithmetic mean concentration in each area on each day was calculated. When only the sum of ^134^Cs and ^137^Cs concentrations was available, individual concentrations were estimated from the average ratio of ^134^Cs to the sum of ^134^Cs and ^137^Cs, namely 0.49 (March–June 2011), 0.46 (July–September 2011), 0.45 (October–December 2011), or 0.42 (January–March 2012), as calculated from foods in which both the ^134^Cs and the ^137^Cs concentration exceeded 25 Bq/kg [1]. The radionuclide concentrations in crude tea and manufactured tea were converted to those in tea leaf by multiplying by the weight ratio of crude tea to tea leaf (0.22) [30]. The concentrations of ^131^I in foods on days before monitoring began or for which there were no data were approximated from the concentrations measured on the closest day and from the half-life of ^131^I (8.04 d [4]). The concentrations of ^134^Cs and ^137^Cs on days before monitoring began or for which there were no data were regarded as the same as the concentrations measured on the closest day. The consumption dates and concentrations in foods were assumed to be as reported [1], [3].

#### Arrival shares

Since citizens generally purchase food from markets, which are supplied from the whole country, the arrival share is an important parameter for estimating the doses due to ingestion. A few citizens in Fukushima City consumed vegetables produced in local fields or home gardens (4.5% of workers in Fukushima City were agricultural workers [31]). We therefore considered two scenarios: Case 1, citizens consumed foods from markets; Case 2, citizens consumed vegetables produced in Fukushima Prefecture and other foods from markets. Case 2 means the arrival shares of vegetables from Fukushima Prefecture are 100%, so this scenario can be regarded as conservative. For citizens in Tokyo and Osaka, only Case 1 was considered. The proportions of targeted foods produced in each area for the markets in each city were calculated as follows. The arrival shares of vegetables and fisheries products were based on the amounts arriving from each prefecture at each central wholesale market in 2010 [26], [32], [33]. We used the 2010 data so as to evaluate the effects of countermeasures. Since data on the importation of fisheries products from overseas were not available, we used the Japanese food self-sufficiency rates based on quantities of production, imports, and exports [17]. The production of marine products in Fukushima Prefecture was regarded to be nil owing to the tsunami damage and the suspension of fishing. The arrival shares of beef, pork, and chicken eggs were based on the quantities transported from each prefecture to Fukushima, Tokyo, and Osaka prefectures [34], [35]. The arrival shares of beef and pork were corrected to the food self-sufficiency rates of Japan. The arrival shares of milk and dairy products were based on the production and transport of raw milk in each prefecture, the amount of milk transported from each prefecture to Fukushima, Tokyo, and Osaka prefectures [36], and the food self-sufficiency rates. For rice, other grains, tea, soybeans, and chicken, we used the nationwide production rates [30], [35], [37], [38] and the food self-sufficiency rates of Japan. The use of these sources is reasonable, especially for dried foods. To estimate the ratios of restricted food production among the prefectural sub-groups, we used the number of cows in each municipality for milk and dairy products [39], the cultivated areas of vegetables and tea [40], the areas of rice paddies [40] and the number of rice farm households [3], and the production [41] or the number of management bodies [42] for shiitake mushrooms.

#### Calculation periods

It is likely that rainfall on the night of 15–16 March 2011 [43] caused heavy contamination of leafy vegetables and of milk and dairy products by radionuclides, so we estimated intake via these products before the first release of data (19 March 2011): from 17 March 2011 in Fukushima City, on the assumption that delivery to citizens takes 1 day; and from 18 March 2011 in Tokyo and Osaka, on the assumption that it takes 2 days. The intake of other foods was estimated from 21 March 2011, when the Japanese government restricted the distribution of foods. Since radionuclide contamination in other foods was not attributable to direct deposition [22], the intake before 21 March was not included. This was supported by the lower contamination of other foods by ^131^I and ^134^Cs and ^137^Cs than of leafy vegetables (Figures S2, S3). The intake was estimated from these days to 20 March 2012.

### Effects of countermeasures

We evaluated the effects of countermeasures (restrictions on the distribution of foods, voluntary withholding of rice, and distribution of bottled water for infants) on reducing intake. We assumed the consumption of foods harvested in the areas where distribution was restricted or rice was withheld to be nil: citizens ate alternative foods containing negligible radionuclides. In this regard, however, for Case 2, the conservative scenario, we considered the effects of restrictions on the distribution of other foods except vegetables, because we assumed that some citizens consumed vegetables grown locally. Since the Tokyo Metropolitan Government distributed bottled water for infants on the morning of 24 March, we evaluated the effect of this countermeasure on 24 and 25 March. (Fukushima City distributed bottled water on the night of 11 March following the earthquake, but this was too early to consider).

### Variations in individual doses

The variations in ingestion doses for ≥19-y-old males (with countermeasures in place) were estimated by Monte Carlo simulation using the commercially available software Crystal Ball (Oracle, California, USA). The drinking water and food variation data input into the Monte Carlo simulation were obtained as below.

We took into account variations in daily consumption of drinking water [44] (Table S2). The drinking water in Tokyo is derived from several sources: 38.0% of tap water comes from the Edogawa River, 41.3% from the Arakawa River, 14.4% from the Tamagawa River, and 6.3% from other sources such as ground water [45], [46]. Therefore, we also took into account the radionuclide concentrations in each source. We assumed that citizens drank water from a single source. The radionuclide concentration factors were estimated from the ratios of ^131^I concentrations in representative purification plants (Kanamachi purification plant for the Edogawa River, Asaka purification plant for the Arakawa River, Ozaku purification plant for the Tamagawa River) to those in tap water in Shinjuku [2], [46]. We used the arithmetic mean concentrations on 22–28 March in the purification plants and on 23–29 March in Shinjuku, on the assumption that delivery to the tap takes 1 day. Concentrations were corrected for half-life. Concentration factors were 3.51 for the Edogawa River, 1.31 for the Arakawa River, and 0.46 for the Tamagawa River (i.e., the ^131^I concentration in the Ozaku purification plant on the Tamagawa River was lower than that in tap water in Shinjuku). The ^131^I concentration in other water systems was regarded as negligible. Fukushima City has only one drinking water treatment plant, and radionuclides were not detected in the drinking water in Osaka. We therefore did not need to take into account variations in the water resources in these two cities.

For input into the Monte Carlo simulation, variations in the dose from drinking water in Fukushima City were estimated from the variation in daily consumption under the assumption of a log-normal distribution. Variations in Tokyo were estimated from variations in daily consumption, the dependence on water source, and the concentration factor.

Variations in the dose from foods for input into the model were estimated under the assumption that citizens bought each food once a week, and the production area was randomly selected according to the distribution of the arrival shares. The weekly average concentrations of radionuclides in each food were calculated. The variations in the daily consumption of each food were as reported [28], [47]. Since these values are based on 1-day surveys, not long-term surveys, their use could have caused us to overestimate the results. The variations in doses obtained by ingesting each food from the same area in the same week at the same daily rate of consumption were considered to indicate variations in the radionuclide concentrations. The relative standard deviations of the variations were estimated from the effective doses calculated from data measured on the same day in Tochigi Prefecture for “ging-geng-cai,” in Ibaraki Prefecture for “marine products,” in Shizuoka Prefecture for “tea,” and in Fukushima Prefecture for other foods (*n*≥4 for each food; Table S2). Daily consumption of foods and the radionuclide concentrations in foods were assumed to follow a log-normal distribution. The variations in the dose from each food were estimated from the input data for the Monte Carlo simulation, namely, the variation in daily consumption, the distribution of arrival shares, and the variation in doses received by ingesting the foods in the same areas. The simulation was performed 10 000 times for drinking water and for each food.

### Estimation of cancer risk

The lifetime attributable risks (LARs) of cancer incidences up to the age of 89 y were estimated in accordance with the method described in a WHO report [48] and Harada et al. [49]. We estimated those due only to the ingestion of ^131^I, ^134^Cs, and ^137^Cs for citizens in each city and those due to three pathways–ingestion, inhalation in the radioactive cloud, and external exposure to material deposited on the ground and in the cloud–for citizens in Fukushima City. Estimation of doses due to inhalation and external exposure followed previous references [15], [50] as described in the Supporting Information. The risk models were based on data from survivors of the atomic bombing in Japan; the validity of the thyroid cancer incidence was also confirmed by a cohort study performed after the Chernobyl accident [51] in the WHO report [48]. A linear-quadratic dose-response model was used for leukemia [52], and linear non-threshold (LNT) models were used for all solid cancers, breast cancer, and thyroid cancer [53]. LAR was calculated from cancer-free survival rates (*S*(*a*, *g*)), an excess absolute risk (EAR) model, and an excess relative risk (ERR) model, as follows: 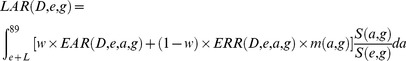
(3)where *D* is the annual dose (Sv), *e* is the age at exposure, *g* is sex, *L* is the minimum latency period, *w* is the weight of the EAR and the ERR models combined, *a* is the age attained, and *m*(*a*, *g*) is the baseline cancer incidence rate in the unexposed population.

The organ (colon, bone marrow, breast, and thyroid) doses were calculated from the effective doses and the organ dose-to-effective dose ratios for three age groups (<1 y, 10 y, and 20 y in the first year) and for the two sexes in the three cities [48]. Doses in the second and subsequent years were calculated from the doses of ^134^Cs and ^137^Cs in March 2012 and from the physical decay of ^134^Cs and ^137^Cs (half-lives of 2.06 and 30 y, respectively [4]).

The cancer-free survival rates of males and females were derived from the age- and sex-stratified all-cause mortality in Japan in 2010 [54] plus the difference between the all-cancer incidence in Japan in 2008 [55], [56] and the all-cancer mortality in Japan in 2010 [54]. Cancer incidences in Japan in 2008 [55], [56] were used as baseline incidence rates for all solid cancers, leukemia, breast cancer, and thyroid cancer.

The minimum latency period was set at 2 y for leukemia, 3 y for thyroid cancer, and 5 y for breast and all solid cancers. The weights of the EAR model and the ERR model combined were set at 0.5 for all solid cancers, leukemia, and thyroid cancer, and 1 for breast cancer.

The details of the EAR and ERR models and their parameters for leukemia, all solid cancers, breast cancer, and thyroid cancer are described in the Supporting Information.

The relationship between cancer risk and dose is still uncertain [57]. However, for the same dose, the risks at low dose rates are known to be lower than those at high dose rates [58]. In particular, coordination of DNA repair processes plays a critical role in allowing proper development and survival of organisms [59].To correct the risk at low dose levels, the International Commission on Radiological Protection (ICRP) and the BEIR VII committee adopted “dose and dose-rate effectiveness factors” of 2 [60] and 1.5 [61]. We followed the ICRP lead and set a factor of 2 for LNT models from the perspective of radiological protection.

### Sources of uncertainty

This study included sources of uncertainty: ^134^Cs and ^137^Cs concentrations in tap water in Fukushima City, limited data on foods in the early stages, data that were less than detection limits, individual behaviors (such as purchasing of bottled water or not purchasing products from Fukushima Prefecture), and assessment of doses in the second and subsequent years for cancer estimation.

^134^Cs and ^137^Cs concentrations in tap water in Fukushima City were estimated from the ^131^I concentrations and the ratios of ^134^Cs and ^137^Cs to ^131^I in tap water in Tokyo by assuming that the ratios of ^134^Cs and ^137^Cs deposition to ^131^I deposition and the removal efficiencies in drinking water treatment plants were similar between Fukushima City and Tokyo. The deposition ratios are known to be similar in the two cities [62], and drinking water treatment plants in both cities use sedimentation and rapid sand filtration, which are effective for removing ^134^Cs and ^137^Cs [63]. This uncertainty did not have a large effect, because the contributions of ^134^Cs and ^137^Cs from drinking water were minor (see “Comparison of doses among ages, effects of countermeasures, and change of intake over time).

In the early stages of monitoring, data were not available for some foods in some prefectures in the Kanto region, but these contributions were judged to be small (see Supporting Information). The number of samples was limited for some foods in the early stages even in Fukushima Prefecture. We therefore used a Monte Carlo simulation and show the variations in dose (e.g., 95th percentile value), including those resulting from radionuclide concentrations, in foods in the early stages.

We regarded radionuclide concentrations that were less than detection limits as nil. There were differences in detection limits among periods and institutions surveyed. We therefore confirmed the results through validation against observations in market basket, food-duplicate, and whole-body-counter surveys.

Differences in individual behaviors, such as not purchasing products from Fukushima Prefecture, could have influenced the variation in estimates. However, as the volume of major crops shipped from Fukushima Prefecture did not decrease after the accident [64], [65], we ignored differences in behaviors. The nationwide average of bottled soft drink consumption was 410 g/d in 2011 [66], and some citizens also purchased bottled water so as to avoid tap water. We ignored the consumption of bottled water and soft drinks: the assumption that people drank only tap water would conservatively overestimate the dose from drinking water.

The doses due to ingestion in the second and subsequent years for cancer estimation were calculated from the physical decay, although the actual doses might be lower because of tighter regulations. The doses due to external exposure from September 2014 were also calculated from the physical decay. These assumptions can be regarded as conservative.

## Results and Discussion

### Validation of results against market basket and food-duplicate surveys

The effective doses due to ingestion of ^134^Cs and ^137^Cs in the diet in Fukushima City (Case 1), Tokyo, and Osaka agreed within a factor of 2, in general, with those calculated in the market basket [1] and food-duplicate surveys [1], [13], [67] in five periods from July 2011 to March 2012 (Table 1). The market basket survey includes drinking water as well as foods. This good agreement supports the accuracy and reliability of our results. The doses in the diet in Fukushima City (Case 2) were higher than those in the market basket and food-duplicate surveys. This result is reasonable because Case 2 is conservative. Validation for the period from March to June, including for the thyroid equivalent doses due to ^131^I, was not done, because no data from market basket and food-duplicate surveys were available.

**Table 1:** Comparison of effective doses of ^134^Cs and ^137^Cs in the diet between this study and the market basket and food-duplicate surveys (µSv/month).

	Jul.2011	Sep.–Nov. 2011	Dec. 2011	Feb.–Mar. 2012	Mar. 2012
Fukushima City (Case 1; this study)^a^	2.36	1.18	0.96	0.51	0.44
Fukushima City (Case 2; this study)^a^	4.15	2.85	3.08	6.84	2.60
Fukushima Prefecture (ref[67])^b^	0.53 ± 1.04^f^	-	-	-	-
Fukushima Prefecture (ref[1])^c^	-	1.6	-	0.33-0.55	-
Fukushima Prefecture (ref[1])^b^	-	-	-	-	0.18^g^
Fukushima Prefecture (ref[13])^b^	-	-	2.17± 1.67^f^	-	-
Tokyo (this study) a	-	0.44	0.24	0.18	0.19
Tokyo (ref[1])^c^	-	0.22	-	-	-
Kanto (ref[1])^c^^,^ d	-	-	-	0.28-0.33	-
Kanto (ref[1])^b^^,^ d	-	-	-	-	0.15^g^
Kanto (ref[13])^b^^,^ e	-	-	0.92± 1.42^f^	-	-
Osaka (this study)^a^	-	0.15	0.09	0.07	0.07
Osaka (ref[1])^c^	-	-	-	0.13	-
Osaka (ref[1])^b^	-	-	-	-	0.1^g^

Case 1, citizens consumed vegetables bought from markets. Case 2, citizens consumed vegetables grown locally.

a with countermeasure (≥19 y old male).

b food-duplicate survey.

c market basket survey.

d including Saitama, and Kanagawa prefectures.

e including Tochigi, Gunma, Ibaraki, Saitama, Chiba, Tokyo, Kanagawa, and Nagano prefectures.

f arithmetic mean ± standard deviation.

g Mar.-May, 2012.

### Comparison of doses among cities

The average thyroid equivalent doses (and effective doses) due to ingestion of ^131^I for a ≥19-y-old male were 840 µSv (43 µSv) in Fukushima City (Case 1), 2700 µSv (140 µSv) in Fukushima City (Case 2), 370 µSv (19 µSv) in Tokyo, and 16 µSv (0.82 µSv) in Osaka (Table 2). The average effective doses due to ^134^Cs and ^137^Cs were 19, 120, 6.1, and 1.9 µSv, respectively. The average effective doses due to total radionuclides were 62, 260, 25, and 2.7 µSv, respectively. There were no large differences between the average and median (within a factor of <1.5, with the exception of the thyroid equivalent dose due to ^131^I in Osaka). The slightly lower thyroid equivalent dose due to ^131^I for citizens of Tokyo in this study (370 µSv) than in our previous study (410 µSv) [19] is attributable to the modification of daily consumption rates.

**Table 2:** Average doses and variations due to countermeasures resulting from the intake of ^131^I, ^134^Cs and ^137^Cs from drinking water and foods by a ≥19-y-old male from 16 March 2011 to 20 March 2012.

	Average	5th percentile	25th percentile	Median	75th percentile	95th percentile
^131^I (thyroid equivalent dose: µSv)						
Fukushima City (Case 1)	840	360	520	690	940	1700
Fukushima City (Case 2)	2700	940	1500	2200	3300	6200
Tokyo	370	140	290	470	780	1400
Osaka	16	<1	1	4	13	74
						
^134^Cs and ^137^Cs (effective dose: µSv)						
Fukushima City (Case 1)	19	8.1	11	15	22	43
Fukushima City (Case 2)	120	60	86	110	150	220
Tokyo	6.1	3.0	4.4	5.8	7.7	12
Osaka	1.9	0.49	0.88	1.4	2.1	4.5
						
Total (effective dose: µSv)						
Fukushima City (Case 1)	62	29	39	50	66	110
Fukushima City (Case 2)	260	120	170	230	310	530
Tokyo	25	11	21	30	47	80
Osaka	2.7	0.59	1.2	1.8	3.0	7.1

Case 1, citizens consumed vegetables bought from markets. Case 2, citizens consumed vegetables grown locally.

The dose due to ingestion of ^131^I in Case 2 was 3 times that in Case 1 and the dose due to ^134^Cs and ^137^Cs was 6 times that in Case 1. The doses in Fukushima City (Case 1) were lower than the values reported in the WHO preliminary assessment (adults in Fukushima City, thyroid equivalent dose, 800–8000 µSv; effective dose due to total radionuclides, 500–5000 µSv) [15] and the UNSCEAR report (absorbed dose to thyroid, 7800 µGy; effective dose due to total radionuclides, 900 µSv) [16]. Regional trade in foods was considered in this study, whereas the WHO preliminary assessment and UNSCEAR report assumed that consumers consumed mainly food produced in Fukushima and neighboring prefectures. Inclusion of regional trade in foods is a key to accurate dose assessment.

**Figure 1:**
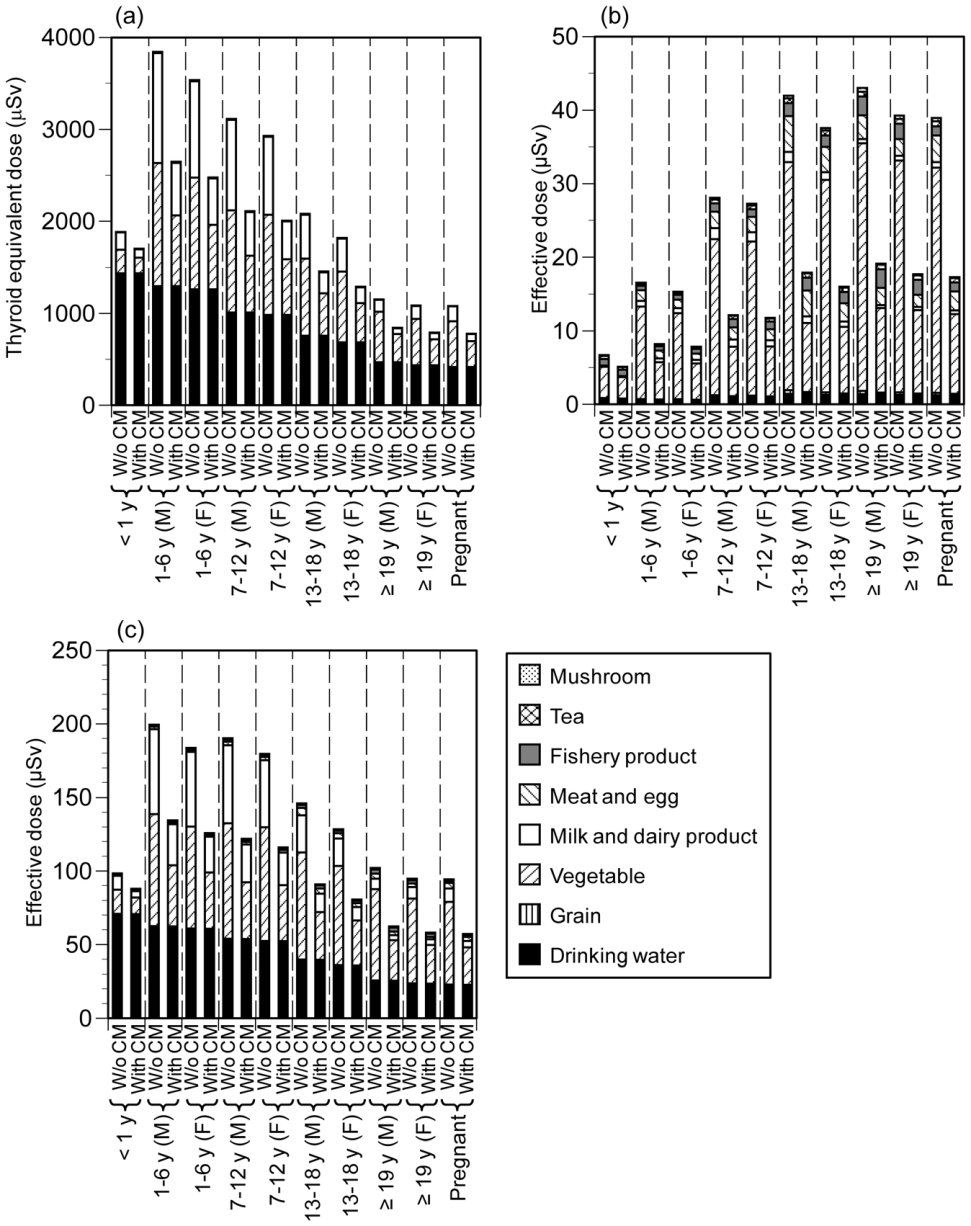
Average doses with and without countermeasures in Fukushima City (Case 1): (a) ^131^I, (b) ^134^Cs and ^137^Cs, (c) total. CM, countermeasures; M, male; F, female. Case 1, citizens consumed vegetables bought from markets.

In both doses, Fukushima City had the highest values and Osaka the lowest. This was consistent with the distances from the nuclear power plant (i.e. Fukushima City, ∼50 km; Tokyo, ∼230 km; Osaka, ∼580 km). The contribution of effective dose from ^131^I to total radionuclides was higher than that from ^134^Cs and ^137^Cs in Fukushima City and Tokyo, and lower in Osaka. Except in Case 2, drinking water contributed the highest thyroid equivalent dose due to ingestion of ^131^I in Fukushima City and Tokyo, followed by vegetables (Figures 1, S5, and S6). ^131^I was not detected in drinking water in Osaka, and vegetables contributed the highest effective dose due to ^134^Cs and ^137^Cs (Figure S7). These results indicate that local contamination of drinking water caused higher intake of ^131^I by citizens in Fukushima City and Tokyo than in Osaka, and that regional trade in foods played an important role in the intake of ^134^Cs and ^137^Cs by citizens in Osaka.

The 95th percentile effective dose due to ingestion of ^134^Cs and ^137^Cs in Fukushima City (Case 1) was 43 µSv; this was 4 times the 97th percentile internal exposure to ^134^Cs and ^137^Cs (i.e., 10 µSv, the value below which the exposures of 328 out of 337 individuals fell) in inhabitants of the village of Kawauchi in Fukushima Prefecture, as monitored by whole-body-counter surveys in November 2011 [12]. The effects of differences in monitoring time were small, because our average effective dose due to ^134^Cs and ^137^Cs in November 2011 (1.3 µSv/month) was similar to the annual average (19 µSv/y = 1.6 µSv/month). Note that the whole-body-counter surveys had sampling bias and may have underestimated the doses received by the general population [11]. The 95th percentile effective doses due to total radionuclides were 110 µSv in Fukushima City (Case 1), 530 µSv in Fukushima City (Case 2), 80 µSv in Tokyo, and 7.1 µSv in Osaka, 2 to 3 times the average values (Table 2). These values were much lower than the annual effective dose due to other natural radionuclides in the diet; e.g., potassium 40 (^40^K), 130–217 µSv [68]; polonium 210 (^210^Po), 730 µSv [69]. The provisional limits of radionuclides in drinking water and foods, which were announced by the Japanese government just after the accident, were determined from the intervention levels of 50 000 µSv of an annual thyroid equivalent dose due to ^131^I and 5000 µSv of an annual effective dose due to ^134^Cs, ^137^Cs, ^89^Sr, and ^90^Sr [22]. Standards released in April 2012 were determined from the intervention level of 1000 µSv of annual effective dose [21]. The 95th percentile doses due to ingestion of individual and total radionuclides in Fukushima City (Cases 1 and 2) and Tokyo were greater than or equal to 1 order of magnitude lower than the provisional limits and also lower than the new limits. Those in Osaka were greater than 2 orders of magnitude lower.

### Comparison of doses among ages, effects of countermeasures, and change of intake over time

The thyroid equivalent dose due to ingestion of ^131^I in Fukushima City (Case 1) decreased with increasing age from 1 y (Figure 1, Tables S5, S7), although the daily consumption of drinking water and most foods increases with age (Table S2). This discrepancy between dose and daily consumption is attributable to the thyroid ingestion dose coefficient, which depends largely on age. In contrast, the effective doses due to ^134^Cs and ^137^Cs increased with age. Overall, the effective doses due to total radionuclides were higher for younger people except for <1-y-old infants (Table S6, S8). The difference in dose between sexes was small. Because the dose coefficients are the same for males and females (Table S1), the differences in doses can be attributed to differences in consumption patterns. Similar results were found in Case 2, Tokyo and Osaka, with the exception that there were no large differences in effective dose due to total radionuclides among ages in Osaka (Figures S5–S7, Tables S9–S20).

The countermeasures reduced the intake of ^131^I by >1-y-olds by 27%–32% in Fukushima City (Case 1), by 11%–15% in Tokyo, and by 33%–37% in Osaka, and reduced the intake of ^134^Cs and ^137^Cs by 49%–57% in Fukushima City (Case 1), by 18%–25% in Tokyo, and by 16%–24% in Osaka (Table S21). The reductions of intake were generally lower for <1-y-old infants because the contributions of drinking water were higher for them.

**Figure 2:**
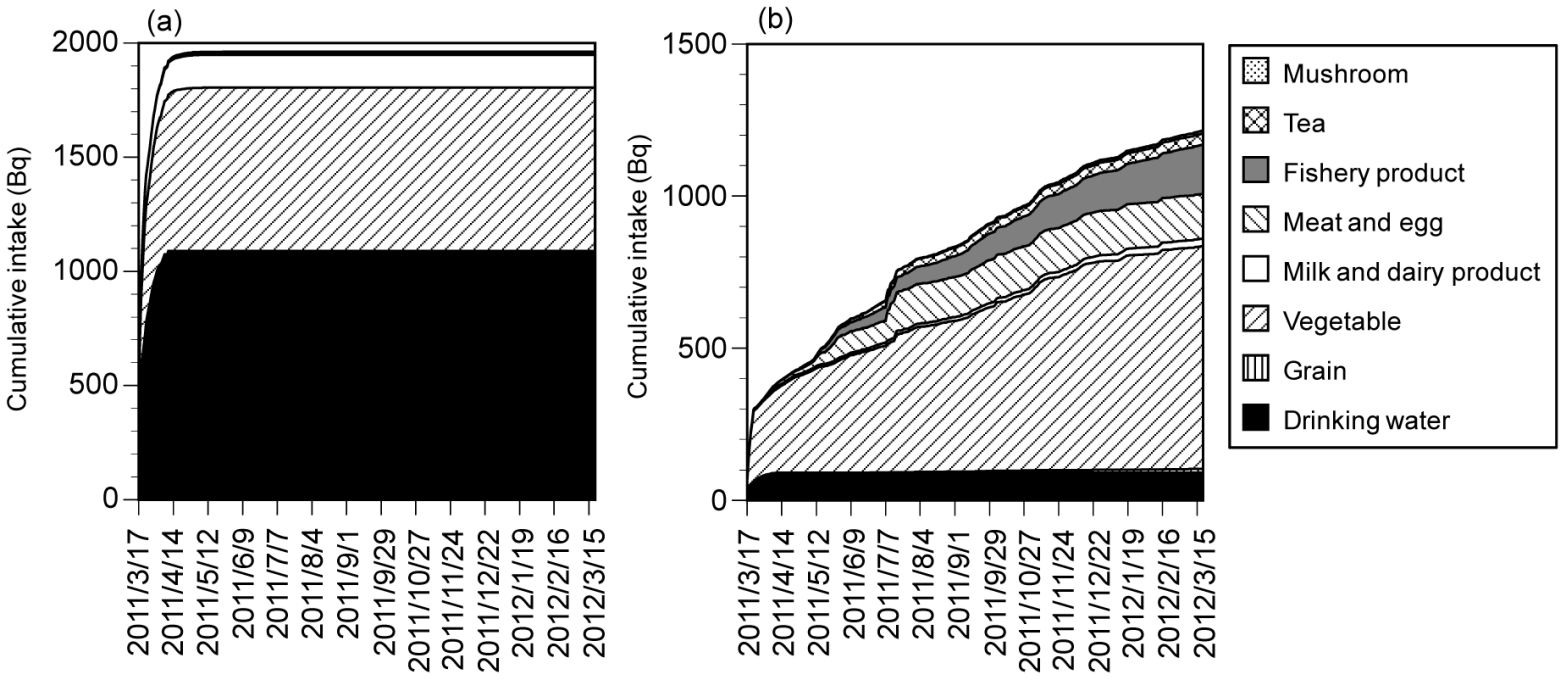
Cumulative intakes for ≥19-y-old male in Fukushima City (Case 1) with countermeasures: (a) ^131^I, (b) ^134^Cs and ^137^Cs. Case 1, citizens consumed vegetables bought from markets.

The cumulative intakes of ^131^I for ≥19-y-old males in Fukushima City (Case 1) in the presence of countermeasures were 72% of the total in week 1 and 87% in week 2 (Figure 2) and stopped increasing within 1 month. This was consistent with our previous result [19] and is attributable to the short half-life of ^131^I and the absence of new serious releases from the nuclear power plant to the atmosphere. The intake of ^131^I was dominant within the first 2 weeks: rapid countermeasures are therefore important in reducing intake. On the other hand, the cumulative intakes of ^134^Cs and ^137^Cs were 25% of the total in week 1 and 27% in week 2. The longer-term intake of ^134^Cs and ^137^Cs is due to the longer half-lives. In particular, the intake via consumption of vegetables and fisheries products was continuous. Similar results were found in Case 2, Tokyo and Osaka (Figures S8–S10).

### LARs of cancer incidence

The LARs of cancer from ingestion are summarized in Table 3. These values do not include risks from external exposure and inhalation. The LARs of all the solid cancer risks were 8.4–12×10^−6^ in Fukushima City (Case 1), 45–71×10^−6^ in Fukushima City (Case 2), 3.0–5.0×10^−6^ in Tokyo, and 1.1–2.0×10^−6^ in Osaka, whereas the LARs of leukemia were >1 order of magnitude lower than those for all the solid cancers (i.e., 0.34–0.59×10^−6^ in Fukushima City (Case 1), 2.0–3.5×10^−6^ in Fukushima City (Case 2), 0.14–0.25×10^−6^ in Tokyo, and 0.05–0.09×10^−6^ in Osaka). The risk of all solid cancers combined together with leukemia is intended to provide an overall indication of the lifetime risk of cancer; however, in circumstances where the tissue doses are highly heterogeneous, such as with doses of ^131^I to the thyroid, the risk of all solid cancers underestimates the cancer risk in specific tissues [48]. This is because the LARs of thyroid cancer in some young groups (<1 y and 10 y in the first year) in Fukushima City and Tokyo exceeded those of all solid cancers. The LARs of all solid cancers estimated from the colon dose are not suitable in the case of ingestion, although they may be still useful as indicators of the risk of deadly cancers. The LARs of thyroid cancers were 2.3–39×10^−6^ in Fukushima City (Case 1), 10–98×10^−6^ in Fukushima City (Case 2), 0.95–14×10^−6^ in Tokyo, and 0.11–1.3×10^−6^ in Osaka, and the maximum LAR in Fukushima City (Case 1) was found in females <1 y old. These values were calculated from the average doses. The 95th percentile doses were 2× the average values for Fukushima City (Cases 1 and 2) and 3× those for Tokyo and Osaka. Because of the variation of doses, a factor of at least 2–3 could be applied to the 95th percentiles of LARs. In addition, these values cannot be directly compared with the ongoing results of diagnosis of thyroid cancers in Fukushima Prefecture because of the screening effects of diagnosis and the use of advanced ultrasound techniques [18]. The contributions of ^131^I to the lifetime-exposure thyroid doses were 92%–96% in Fukushima City (Case 1), 84%–92% in Fukushima City (Case 2), 91%–96% in Tokyo, and 62%–78% in Osaka. As described above, most of the ^131^I was taken up within the first 2 weeks. The LARs of thyroid cancers were attributed mainly to ^131^I in the first 2 weeks, highlighting again the fact that rapid implementation of countermeasures after a nuclear accident is important. On the other hand, Japanese dietary exposure gives lifetime cancer risks of 310×10^−6^ from inorganic arsenic (for the sum of skin, liver, and lung cancers) [70] and 1400×10^−6^ from acrylamide [71]. Cancer risks from ingestion–especially in the case of the thyroid cancer risk in young females in Fukushima City–might not be negligible, but they are 1 to 2 orders of magnitude lower than the cancer risks from ubiquitous carcinogens in the daily diet.

**Table 3:** LARs for all solid cancers, leukemia, breast cancer, and thyroid cancer (×10^−6^) due to ingestion.

	All solid cancers	Leukemia	Breast cancer	Thyroid cancer
Fukushima City (Case 1)				
<1 y (M)	8.6	0.57	-	9.5
<1 y (F)	12	0.36	1.5	39
10 y (M)	8.4	0.59	-	7.6
10 y (F)	11	0.36	1.5	30
20 y (M)	7.4	0.56	-	2.3
20 y (F)	9.8	0.34	1.1	9.0
Fukushima City (Case 2)				
<1 y (M)	47	3.1	-	15
<1 y (F)	66	2.0	8.4	62
10 y (M)	51	3.5	-	25
10 y (F)	71	2.2	9.3	98
20 y (M)	45	3.4	-	10
20 y (F)	61	2.1	7.1	38
Tokyo				
<1 y (M)	3.6	0.24	-	3.4
<1 y (F)	5.0	0.15	0.63	14
10 y (M)	3.6	0.25	-	3.1
10 y (F)	4.7	0.15	0.61	12
20 y (M)	3.0	0.23	-	0.95
20 y (F)	3.9	0.14	0.44	3.6
Osaka				
<1 y (M)	1.4	0.09	-	0.15
<1 y (F)	2.0	0.06	0.25	0.62
10 y (M)	1.3	0.09	-	0.20
10 y (F)	1.8	0.06	0.23	1.3
20 y (M)	1.1	0.08	-	0.11
20 y (F)	1.4	0.05	0.16	0.42

Ages represent ones in the first year. Case 1, citizens consumed vegetables bought from markets. Case 2, citizens consumed vegetables grown locally.

The total effective doses due to ingestion, inhalation, and external exposure in the first year and over a lifetime (up to 89 y) for Fukushima City are summarized in Table S22. The LARs of all solid cancers due to the three pathways were 1400–4100×10^−6^ in Fukushima City (Case 1) and 1400–4200×10^−6^ in Fukushima City (Case 2); those of thyroid cancer were 29–490×10^−6^ in Fukushima City (Case 1) and 37–520×10^−6^ in Fukushima City (Case 2) (Table 4). The contributions of LARs of all solid cancer due to ingestion were 0.3%–0.5% (Case 1) and 1.6%–3.1% (Case 2); those of thyroid cancer were 7.5%–12% (Case 1) and 12%–30% (Case 2). Even when the 95th percentile values were 2× the average, the contributions of LARs of all solid cancers due to ingestion were minor. However, for the 95th percentile values in Case 2, the contribution of LARs of thyroid cancer due to ingestion was approximately half of the total. Except in such extreme situations, the contributions of LARs of thyroid cancer due to ingestion cannot be regarded as either negligible or predominant but as minor.

**Table 4:** LARs for all solid cancers, leukemia, breast cancer, and thyroid cancer (×10^−6^) due to external exposure, inhalation, and ingestion.

	All solid cancers	Leukemia	Breast cancer	Thyroid cancer
Fukushima City (Case 1)				
<1 y (M)	2700	300	-	120
<1 y (F)	4100	210	1200	490
10 y (M)	2000	170	-	66
10 y (F)	3000	110	690	270
20 y (M)	1400	120	-	29
20 y (F)	2100	79	390	120
Fukushima City (Case 2)				
<1 y (M)	2700	310	-	130
<1 y (F)	4200	210	1200	520
10 y (M)	2100	170	-	83
10 y (F)	3100	120	700	340
20 y (M)	1400	120	-	37
20 y (F)	2100	81	390	150

Ages are those in the first year. Case 1, citizens consumed vegetables bought from markets. Case 2, citizens consumed vegetables grown locally.

As described above, the contributions of ^131^I to the lifetime-exposure thyroid doses were >84% for Fukushima City, and the contributions of LARs from foods in the second and subsequent years were negligible. However, in Japan and other countries, some people are still concerned about artificial radionuclides in the diet. Although a greater understanding of risks alone may not lead to reasonable countermeasures, the doses and cancer risks calculated in this study are essential information.

## Supporting Information

Figure S1 **Locations of each prefecture in Japan.**
(PDF)

Figure S2 **^131^I concentrations in leafy vegetables, fruit vegetables, milk and dairy products, meat and eggs, and marine products in Fukushima Prefecture in March 2011.** a: Gunma; b: April.
(PDF)

Figure S3 **^134^Cs and ^137^Cs concentrations in foods in Fukushima Prefecture in 2011.** a: August; b: July; c: June; d: March; e: Gunma in March; f: March–April; g: May; h: April; i: October; j: September; k: Tochigi; December; l: April–May; m: Ibaraki in May.
(PDF)

Figure S4 **Radionuclide concentrations in each prefecture in March 2011.** (a) ^131^I; (b) ^134^Cs and ^137^Cs.
(PDF)

Figure S5 **Average doses with and without countermeasures in Fukushima City (Case 2): (a) ^131^I, (b) ^134^Cs and ^137^Cs, (c) total.** CM, countermeasures; M, male; F, female. Case 2, citizens consumed vegetables grown locally.
(PDF)

Figure S6 **Average doses with and without countermeasures in Tokyo: (a) ^131^I, (b) ^134^Cs and ^137^Cs, (c) total.** CM, countermeasures; M, male; F, female.
(PDF)

Figure S7 **Average doses with and without countermeasures in Osaka: (a) ^131^I, (b) ^134^Cs and ^137^Cs, (c) total.** CM, countermeasures; M, male; F, female.
(PDF)

Figure S8 **Cumulative intakes for ≥ 19 y old male in Fukushima City (Case 2) with countermeasures: (a) ^131^I, (b) ^134^Cs and ^137^Cs.** Case 2, citizens consumed vegetables grown locally.
(PDF)

Figure S9 **Cumulative intakes for ≥ 19 y old male in Tokyo with countermeasures: (a) ^131^I, (b) ^134^Cs and ^137^Cs.**
(PDF)

Figure S10 **Cumulative intakes for ≥ 19 y old male in Osaka with countermeasures: (a) ^131^I, (b) ^134^Cs and ^137^Cs.**
(PDF)

Methods S1 **Numbers of samples analyzed; Dose estimation for inhalation and external exposure; Risk models and their parameters.**
(PDF)

Table S1 **Thyroid equivalent dose coefficients for ingestion of ^131^I and the effective dose coefficients for ingestion of ^131^I, ^134^Cs and ^137^Cs (µSv/Bq).**
(PDF)

Table S2 **Daily consumption rates of drinking water and foods, and relative standard deviations (RSDs) of doses received by ingesting the foods in the same areas.**
(PDF)

Table S3 **Numbers of samples analyzed each month.**
(PDF)

Table S4 **Numbers of samples (leafy vegetables, fruit vegetables, milk and dairy products, meat and eggs, and marine products) analyzed in each prefecture in March and April 2011.**
(PDF)

Table S5 **Average thyroid equivalent doses of ^131^I without countermeasures in Fukushima City (Case 1) in the first year after the accident (µSv).** M, male; F, female. Case 1, citizens consumed vegetables bought from markets.
(PDF)

Table S6 **Average effective doses of ^134^Cs and ^137^Cs without countermeasures in Fukushima City (Case 1) in the first year after the accident (µSv).** M, male; F, female. Case 1, citizens consumed vegetables bought from markets.
(PDF)

Table S7 **Average thyroid equivalent doses of ^131^I with countermeasures in Fukushima City (Case 1) in the first year after the accident (µSv).** M, male; F, female. Case 1, citizens consumed vegetables bought from markets.
(PDF)

Table S8 **Average effective doses of ^134^Cs and ^137^Cs with countermeasures in Fukushima City (Case 1) in the first year after the accident (µSv).** M, male; F, female. Case 1, citizens consumed vegetables bought from markets.
(PDF)

Table S9 **Average thyroid equivalent doses of ^131^I without countermeasures in Fukushima City (Case 2) in the first year after the accident (µSv).** M, male; F, female. Case 2, citizens consumed vegetables grown locally.
(PDF)

Table S10 **Average effective doses of ^134^Cs and ^137^Cs without countermeasures in Fukushima City (Case 2) in the first year after the accident (µSv).** M, male; F, female. Case 2, citizens consumed vegetables grown locally.
(PDF)

Table S11 **Average thyroid equivalent doses of ^131^I with countermeasures in Fukushima City (Case 2) in the first year after the accident (µSv).** M, male; F, female. Case 2, citizens consumed vegetables grown locally.
(PDF)

Table S12 **Average effective doses of ^134^Cs and ^137^Cs with countermeasures in Fukushima City (Case 2) in the first year after the accident (µSv).** M, male; F, female. Case 2, citizens consumed vegetables grown locally.
(PDF)

Table S13 **Average thyroid equivalent doses of ^131^I without countermeasures in Tokyo in the first year after the accident (µSv).** M, male; F, female.
(PDF)

Table S14 **Average effective doses of ^134^Cs and ^137^Cs without countermeasures in Tokyo in the first year after the accident (µSv).** M, male; F, female.
(PDF)

Table S15 **Average thyroid equivalent doses of ^131^I with countermeasures in Tokyo in the first year after the accident (µSv).** M, male; F, female.
(PDF)

Table S16 **Average effective doses of ^134^Cs and ^137^Cs with countermeasures in Tokyo in the first year after the accident (µSv).** M, male; F, female.
(PDF)

Table S17 **Average thyroid equivalent doses of ^131^I without countermeasures in Osaka in the first year after the accident (µSv).** M, male; F, female.
(PDF)

Table S18 **Average effective doses of ^134^Cs and ^137^Cs without countermeasures in Osaka in the first year after the accident (µSv).** M, male; F, female.
(PDF)

Table S19 **Average thyroid equivalent doses of ^131^I with countermeasures in Osaka in the first year after the accident (µSv).** M, male; F, female.
(PDF)

Table S20 **Average effective doses of ^134^Cs and ^137^Cs with countermeasures in Osaka in the first year after the accident (µSv).** M, male; F, female.
(PDF)

Table S21 **Reductions of doses by countermeasures (%).** M, male; F, female. Case 1, citizens consumed vegetables bought from markets.
(PDF)

Table S22 **Effective doses in the first year and over the total lifetime (up to 89 y) due to three pathways (µSv).** Ages are those in the first year. M, male; F, female. Case 1, citizens consumed vegetables bought from markets. Case 2, citizens consumed vegetables grown locally.
(PDF)
