# Correction: Estimated Dietary Intake of Radionuclides and Health Risks for the Citizens of Fukushima City, Tokyo, and Osaka after the 2011 Nuclear Accident

**DOI:** 10.1371/journal.pone.0136223

**Published:** 2015-08-14

**Authors:** Michio Murakami, Taikan Oki

There are errors in the values for LARS for thyroid cancer throughout the paper. Please see below for a description of the errors and their corrections.

There are errors in the last two sentences of the Abstract. The correct sentences are: Lifetime attributable risks (LARs) of thyroid cancers due to ingestion were 1.7–37×10^−6^ (Case 1) and 5.6–79×10^−6^ (Case 2) in Fukushima City, 0.73–13×10^−6^ in Tokyo, and 0.04–0.49×10^−6^ in Osaka. The contributions of LARs of thyroid cancers due to ingestion were 5.4%–11% of all exposure (Case 1) and 11%–25% (Case 2) in Fukushima City.

There are errors in the seventh sentence of the “Lars of cancer incidence” section of the Results and Discussion. The correct sentence is: The LARs of thyroid cancers were 1.7–37×10^−6^ in Fukushima City (Case 1), 5.6–79×10^−6^ in Fukushima City (Case 2), 0.73–13×10^−6^ in Tokyo, and 0.04–0.49×10^−6^ in Osaka, and the maximum LAR in Fukushima City (Case 1) was found in females <1 y old.

There are errors in the eleventh sentence of the “Lars of cancer incidence” section of the Results and Discussion. The correct sentence is: The contributions of ^131^I to the lifetime-exposure thyroid doses were 88%-95% in Fukushima City (Case 1), 80%-91% in Fukushima City (Case 2), 89%–95% in Tokyo, and 42%–67% in Osaka.

There are errors in the second and third sentences of the second paragraph of the “Lars of cancer incidence” section of the Results and Discussion. The correct sentences are: The LARs of all solid cancers due to the three pathways were 1400–4100×10^−6^ in Fukushima City (Case 1) and 1400–4200×10^−6^ in Fukushima City (Case 2); those of thyroid cancer were 29–490×10^−6^ in Fukushima City (Case 1) and 33–510×10^−6^ in Fukushima City (Case 2) (Table 4). The contributions of LARs of all solid cancer due to ingestion were 0.3%–0.5% (Case 1) and 1.6%–3.1% (Case 2); those of thyroid cancer were 5.4%–11% (Case 1) and 11%–25% (Case 2).

There are errors in the first sentence of the last paragraph of the “Lars of cancer incidence” section of the Results and Discussion. The correct sentence is: As described above, the contributions of ^131^I to the lifetime-exposure thyroid doses were >80% for Fukushima City, and the contributions of LARs from foods in the second and subsequent years were negligible.

There are errors in Tables 3 and 4. Please see the corrected Tables 3 and 4 here.

**Table 3:** LARs for all solid cancers, leukemia, breast cancer, and thyroid cancer (×10^−6^) due to ingestion.

	All solid cancers	Leukemia	Breast cancer	Thyroid cancer
Fukushima City (Case 1)				
<1 y (M)	8.6	0.57	-	9.0
<1 y (F)	12	0.36	1.5	37
10 y (M)	8.4	0.59	-	6.9
10 y (F)	11	0.36	1.5	27
20 y (M)	7.4	0.56	-	1.7
20 y (F)	9.8	0.34	1.1	6.4
Fukushima City (Case 2)				
<1 y (M)	47	3.1	-	14
<1 y (F)	66	2.0	8.4	55
10 y (M)	51	3.5	-	20
10 y (F)	71	2.2	9.3	79
20 y (M)	45	3.4	-	5.6
20 y (F)	61	2.1	7.1	21
Tokyo				
<1 y (M)	3.6	0.24	-	3.2
<1 y (F)	5.0	0.15	0.63	13
10 y (M)	3.6	0.25	-	2.9
10 y (F)	4.7	0.15	0.61	11
20 y (M)	3.0	0.23	-	0.73
20 y (F)	3.9	0.14	0.44	2.8
Osaka				
<1 y (M)	1.4	0.09	-	0.08
<1 y (F)	2.0	0.06	0.25	0.32
10 y (M)	1.3	0.09	-	0.12
10 y (F)	1.8	0.06	0.23	0.49
20 y (M)	1.1	0.08	-	0.04
20 y (F)	1.4	0.05	0.16	0.16

Ages represent ones in the first year. Case 1, citizens consumed vegetables bought from markets. Case 2, citizens consumed vegetables grown locally.

**Table 4:** LARs for all solid cancers, leukemia, breast cancer, and thyroid cancer (×10^−6^) due to external exposure, inhalation, and ingestion.

	All solid cancers	Leukemia	Breast cancer	Thyroid cancer
Fukushima City (Case 1)				
< 1 y (M)	2700	300	-	120
< 1 y (F)	4100	210	1200	490
10 y (M)	2000	170	-	65
10 y (F)	3000	110	690	120
20 y (M)	1400	120	-	510
20 y (F)	2100	79	390	78
Fukushima City (Case 2)				
< 1 y (M)	2700	310	-	130
< 1 y (F)	4200	210	1200	520
10 y (M)	2100	170	-	83
10 y (F)	3100	120	700	320
20 y (M)	1400	120	-	33
20 y (F)	2100	81	390	130

Ages are those in the first year. Case 1, citizens consumed vegetables bought from markets. Case 2, citizens consumed vegetables grown locally.
