# Supplementary figures and images for: Estimated Dietary Intake of Radionuclides and Health Risks for the Citizens of Fukushima City, Tokyo, and Osaka after the 2011 Nuclear Accident
Source: PLoS One. 2014 Nov 12;9(11):e112791. doi: 10.1371/journal.pone.0112791 (PMC4229249; doi:10.1371/journal.pone.0112791)

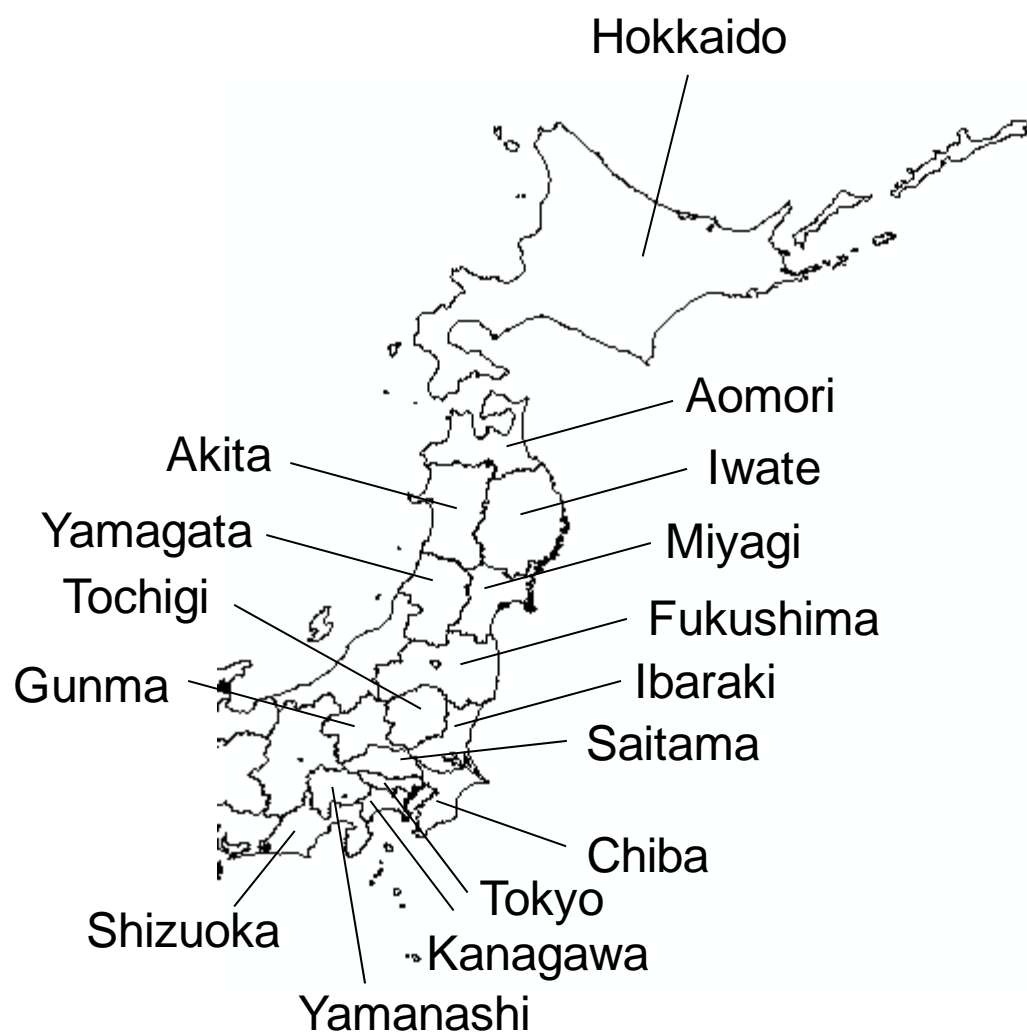

Figure S1. Locations of each prefecture in Japan.

Supplement: Figure S1 — Locations of each prefecture in Japan. (PDF) [file pone.0112791.s001.pdf]

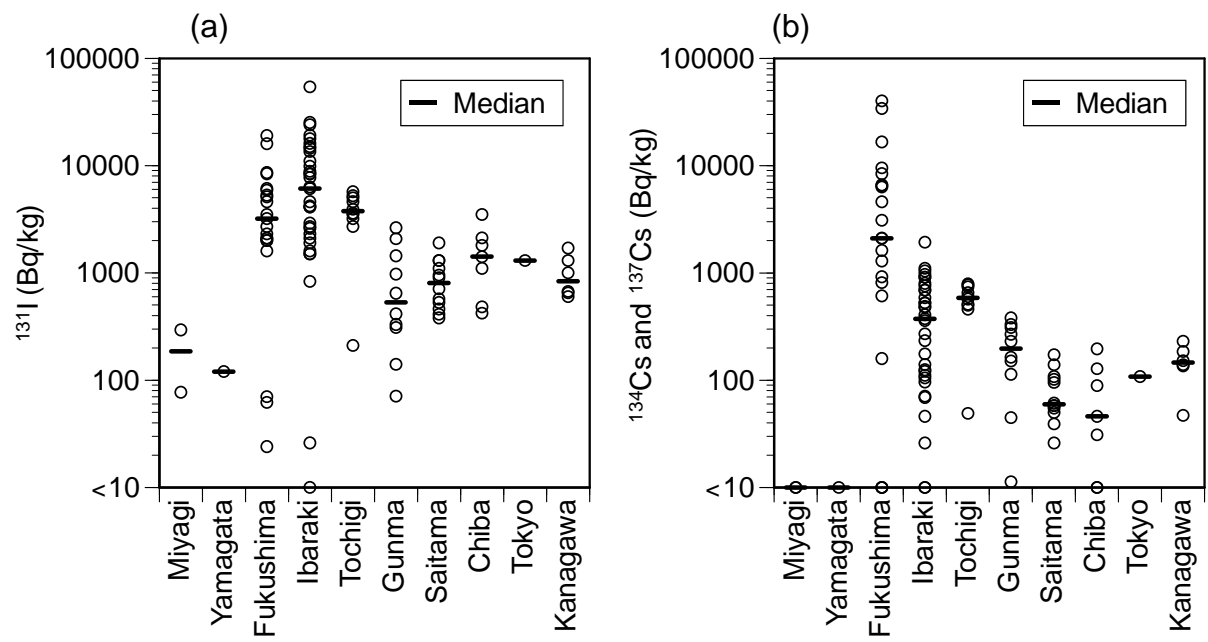

Figure S4. Radionuclide concentrations in each prefecture in March 2011.

(a)  $^{131}\text{I}$ ; (b)  $^{134}\text{Cs}$  and  $^{137}\text{Cs}$ .

Supplement: Figure S4 — Radionuclide concentrations in each prefecture in March 2011. (a) 131I; (b) 134Cs and 137Cs. (PDF) [file pone.0112791.s004.pdf]
