# Supplementary material for: Estimated Dietary Intake of Radionuclides and Health Risks for the Citizens of Fukushima City, Tokyo, and Osaka after the 2011 Nuclear Accident
Source: PLoS One. 2014 Nov 12;9(11):e112791. doi: 10.1371/journal.pone.0112791 (PMC4229249; doi:10.1371/journal.pone.0112791)
Supplement: Figure S2 — 131I concentrations in leafy vegetables, fruit vegetables, milk and dairy products, meat and eggs, and marine products in Fukushima Prefecture in March 2011. a: Gunma; b: April. (PDF) [file pone.0112791.s002.pdf]

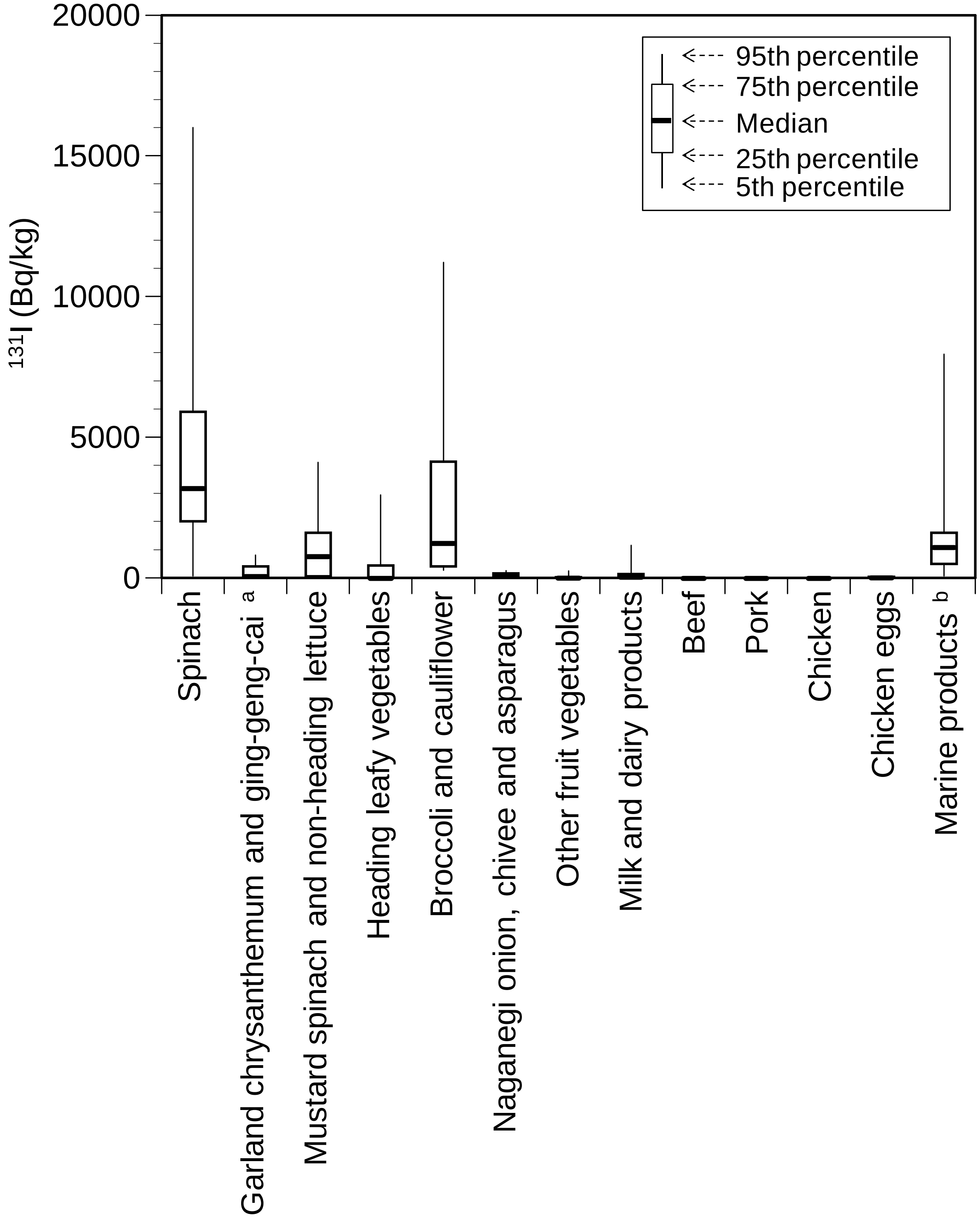

Figure S2. ^{131}I concentrations in leafy vegetables, fruit vegetables, milk and dairy products, meat and eggs, and marine products in Fukushima Prefecture in March 2011. a: Gunma; b: April.
