# Supplementary material for: Estimated Dietary Intake of Radionuclides and Health Risks for the Citizens of Fukushima City, Tokyo, and Osaka after the 2011 Nuclear Accident
Source: PLoS One. 2014 Nov 12;9(11):e112791. doi: 10.1371/journal.pone.0112791 (PMC4229249; doi:10.1371/journal.pone.0112791)
Supplement: Figure S3 — 134Cs and 137Cs concentrations in foods in Fukushima Prefecture in 2011. a: August; b: July; c: June; d: March; e: Gunma in March; f: March–April; g: May; h: April; i: October; j: September; k: Tochigi; December; l: April–May; m: Ibaraki in May. (PDF) [file pone.0112791.s003.pdf]

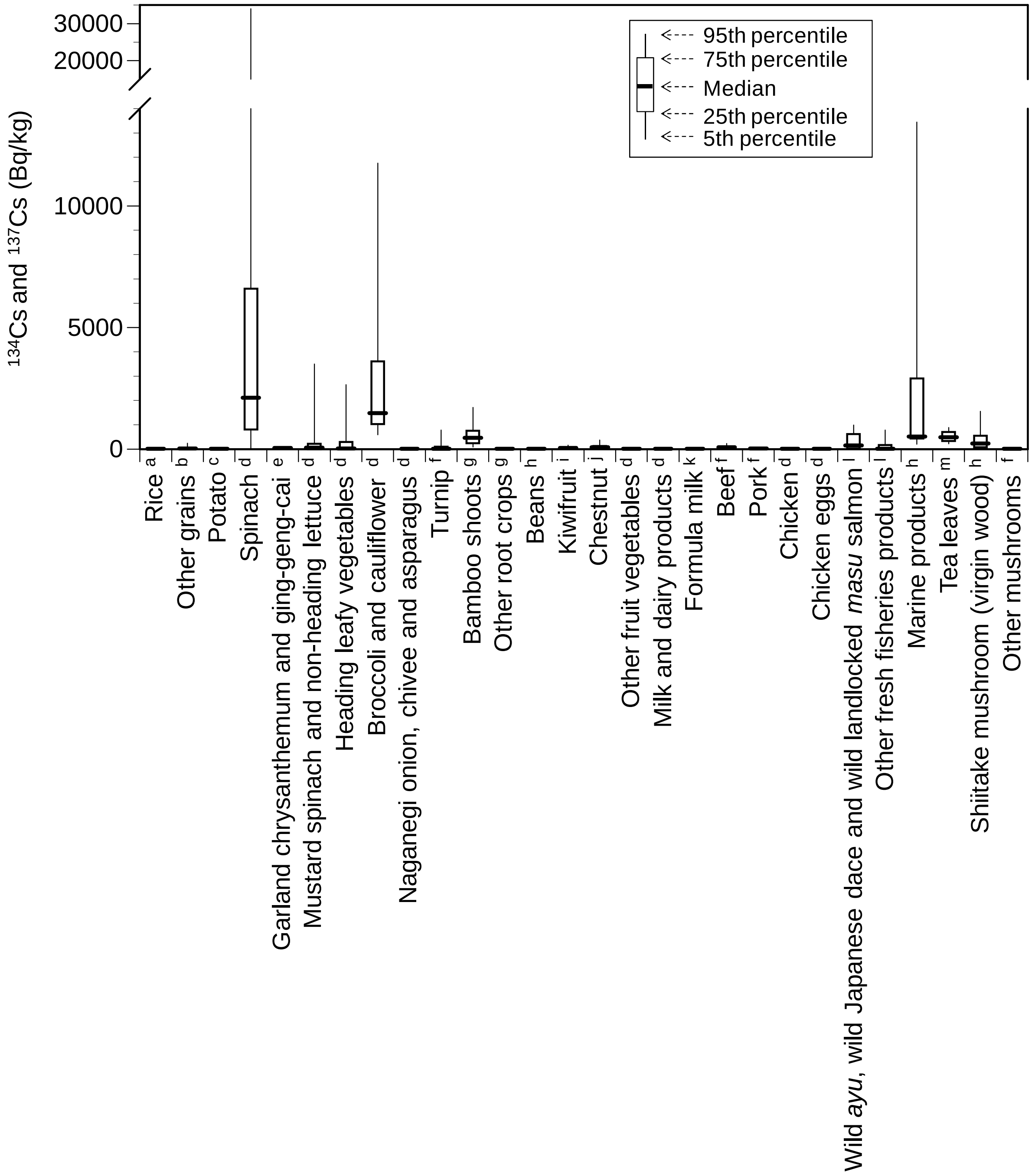

Figure S3. ^{134}Cs and ^{137}Cs concentrations in foods in Fukushima Prefecture in 2011.
a: August; b: July; c: June; d: March; e: Gunma in March; f: March–April; g: May; h: April; i: October; j: September; k: Tochigi; December; l: April–May; m: Ibaraki in May.
