# Supplementary material for: Estimated Dietary Intake of Radionuclides and Health Risks for the Citizens of Fukushima City, Tokyo, and Osaka after the 2011 Nuclear Accident
Source: PLoS One. 2014 Nov 12;9(11):e112791. doi: 10.1371/journal.pone.0112791 (PMC4229249; doi:10.1371/journal.pone.0112791)
Supplement: Figure S5 — Average doses with and without countermeasures in Fukushima City (Case 2): (a) 131I, (b) 134Cs and 137Cs, (c) total. CM, countermeasures; M, male; F, female. Case 2, citizens consumed vegetables grown locally. (PDF) [file pone.0112791.s005.pdf]

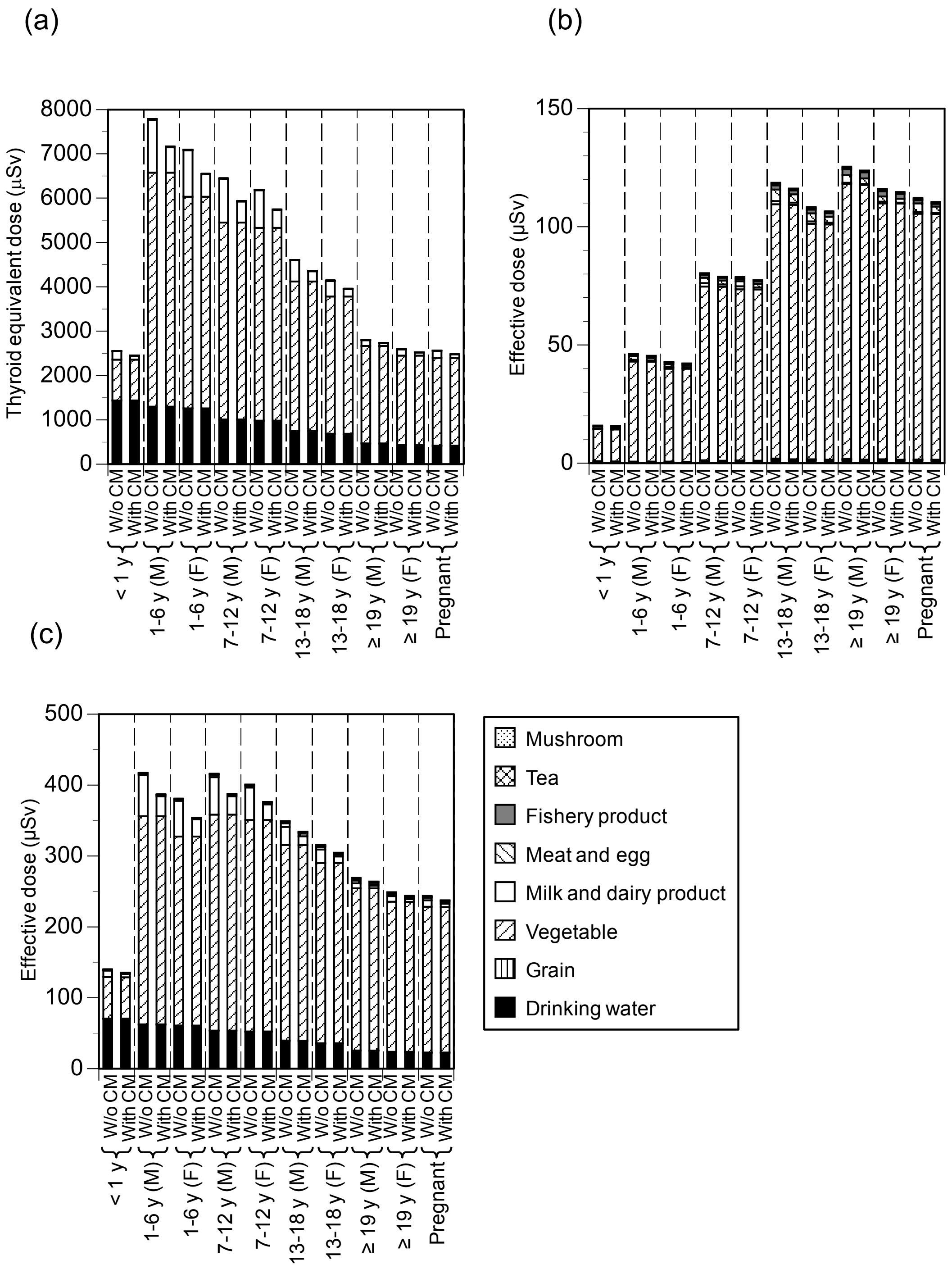

Figure S5. Average doses with and without countermeasures in Fukushima City (Case 2): (a) ^{131}I, (b) ^{134}Cs and ^{137}Cs, (c) total. CM, countermeasures; M, male; F, female. Case 2, citizens consumed vegetables grown locally.
