# Supplementary material for: Estimated Dietary Intake of Radionuclides and Health Risks for the Citizens of Fukushima City, Tokyo, and Osaka after the 2011 Nuclear Accident
Source: PLoS One. 2014 Nov 12;9(11):e112791. doi: 10.1371/journal.pone.0112791 (PMC4229249; doi:10.1371/journal.pone.0112791)
Supplement: Methods S1 — Numbers of samples analyzed; Dose estimation for inhalation and external exposure; Risk models and their parameters. (PDF) [file pone.0112791.s011.pdf]

Methods

Numbers of samples analyzed

The numbers of samples analyzed each month are summarized in Table S3. Food monitoring was started according to production season (e.g. rice: end of summer). The Japanese government extensively monitored leafy vegetables, fruit vegetables, and marine products in the initial months. This was reasonable, because the radionuclide concentrations in leafy vegetables and marine products were much higher than in other foods, as described in the main text and Figures S2 and S3. The numbers of samples analyzed in each prefecture in the initial months are shown in Table S4. Foods were intensively monitored in the Fukushima and Kanto regions. This was also rational, because the radionuclide concentrations in foods in these areas were much higher than those in other prefectures, as described in the main text and shown in Figure S4. However, data were not available for some foods in some prefectures in the Kanto region (e.g. Garland chrysanthemum and ging-geng-cai in Tokyo). Intake of these foods from these areas was regarded as negligible, because the arrival shares were very low (<2%, typically < 0.5%) in most cases, with the following exceptions: mustard spinach and non-heading lettuce from Tochigi to Fukushima, 7%; broccoli and cauliflower from Ibaraki to Tokyo, 4%; pork from Saitama and Chiba to Tokyo, 3% and 11%,

respectively; and chicken eggs from Saitama, Chiba, and Kanagawa to Tokyo, 4%, 13%, and 2%, respectively. We used the radionuclide concentrations in individual foods collected in Fukushima Prefecture as a substitute for the data in these areas to confirm whether the contributions of intakes from these foods from these areas were small. The overall increases in total effective doses were ≤1% and ≤3% for Fukushima and Tokyo, respectively, and we therefore judged that the contributions were small.

Dose estimation for inhalation and external exposure

We estimated the doses due to inhalation in the radioactive cloud and to external exposure to material deposited on the ground and in the cloud for citizens in Fukushima City in order to calculate LARs of cancer incidences due to ingestion, inhalation, and external exposure. Estimations of doses followed previous references[1,2].

The additional effective doses due to inhalation was estimated as[1]:

$$\text{Effective doses due to inhalation } (\mu\text{Sv}) = A_{\text{Cs137}} \times I_i \times (\textstyle\sum_m (A_m/A_{\text{Cs137}})/V_{\text{b}m} \times d_{mi})$$

where m is the radionuclide, i is the population group, A_m is the surface activity density of radionuclide m on the ground (Bq/m^2), I_i is the breathing (inspiration) rate for population group i (m^3/s), the $V_{\text{b}m}$ is the bulk deposition velocity of radionuclide m (m/s), and d_{mi} is the effective dose inhalation coefficient for population group i and

radionuclide m (μSv/Bq). Radionuclides ^{131}I, ^{132}I, ^{132}Te, ^{134}Cs, and ^{137}Cs were considered in the dose estimation. A_{Cs137} was set as the median of measured values (137 000 Bq/m^2[3]) in Fukushima City. V_{bm} was 0.07 m/s for ^{131}I and 0.01 m/s for the other radionuclides.

The additional effective dose due to external exposure was estimated as[2]:

$$\text{Effective dose due to external exposure } (\mu\text{Sv}) = \int (\text{ADR} \times k - \text{BGR})\,\text{dt} \times (t_{\text{out}} + t_{\text{in}} \times \text{RF})/24 \times C_{\text{age}}$$

where ADR is the kerma rate in free air (μGy/h), k is the conversion coefficient from ADR to effective dose (μSv/μGy), BGR is the background rate (0.03 μSv/h)[2], t_{out} is the time spent outdoors in a day (h), t_{in} is the time spent indoors in a day, RF is the reduction factor, and C_{age} is the age coefficient.

ADR was recorded by a radiation monitoring post in the Northern region's health and welfare office in Fukushima City[4] to August 2014. It includes all of the radiation exposure or radioiodine and radiocesium from cloud and ground shine. k was set at 0.75 [1,5]. RF was set at 0.9 for cloud shine before 15 March and 0.4 for ground shine thenceforward[2]. This latter value is comparable to the average value of 0.34 observed in households in Fukushima Prefecture[6]. Values of t_{out} and t_{in} were set at 8 h and 16 h, respectively[1].

The effective doses for children aged <16 years were corrected by multiplying the effective dose estimated above by C_{age}, as follows[2]:

$$C_{age} = -0.0144 \times age + 1.27$$

The effective doses from September 2014 were estimated from the values in August 2014 and the physical decay of ^{134}Cs and ^{137}Cs (half-lives of 2.06 and 30 y, respectively[7]). The radionuclide composition just after the accident was assumed to be ^{134}Cs: ^{137}Cs = 1:1, with a contribution to additional effective dose of ^{134}Cs: ^{137}Cs = 0.73 : 0.27[8]. We did not consider weathering effects or decontamination after September 2014 in the assessment, and therefore the doses can be regarded as conservative.

Risk models and their parameters

The lifetime attributable risks (LARs) of cancer incidences from the ingestion of ^{131}I and ^{134}Cs and ^{137}Cs up to the age of 89 y were estimated in accordance with the method described in a WHO report[9] and Harada et al.[10]. LARs for all solid cancers, leukemia, and thyroid cancer was calculated from combination of an excess absolute risk (EAR) model, and an excess relative risk (ERR) model. LAR for breast cancer was

calculated from the EAR model alone. The risk models and their parameters for leukemia are as follows:

$$\mathrm{EAR}\ (D, e, a, g) = (\alpha \cdot D + \beta \cdot D^2)\ \exp[\kappa_1 \cdot \mathrm{l}_{s=\mathrm{female}} + \kappa_2 \cdot \ln\ (a - e)]$$

where $\alpha = 7.5165 \times 10^{-4}$, $\beta/\alpha = 1.03455$, $\kappa_1 = -0.52526$, $\kappa_2 = -0.6141$, $\mathrm{l}_{s=\mathrm{female}} = 0$ for males, 1 for females.

$$\mathrm{ERR}\ (D, e, a, g) = (\alpha \cdot D + \beta \cdot D^2)\ \exp[\kappa_1 \cdot \ln\ (a)]$$

where $\alpha = 864.552$, $\beta/\alpha = 1.18092$, $\kappa_1 = -1.647$.

Risk models for all solid cancers and thyroid cancer are as follows:

$$\mathrm{EAR}\ (D, e, a, g) \text{ or } \mathrm{ERR}\ (D, e, a, g)$$

$$= (1 + t \cdot s) \cdot k_d \cdot D \exp\ [-g_e \cdot (e\text{-}30) + g_a \cdot \ln(a/70)]$$

where, for the EAR model for all solid cancers, $t = 0.1622$, $k_d = 51.63 \times 10^{-4}$, $g_e = 0.02805$, $g_a = 2.406$; for the ERR model for all solid cancers, $t = 0.2465$, $k_d = 0.4666$, $g_e = 0.01849$, $g_a = -1.621$; for the EAR model for thyroid cancers, $t = 0.5699$, $k_d = 1.232 \times 10^{-4}$, $g_e = 0.05903$, $g_a = 0.5921$; for the ERR model for thyroid cancers, $t = 0.1433$, $k_d = 0.5767$, $g_e = 0.03739$, $g_a = -1.445$; and for all models, $s = -1$ for males and 1 for females.

A risk model for breast cancers is as follows:

$$\text{EAR}\ (D, e, a, g) = k_d \cdot D \exp\ [-g_e \cdot (e\text{-}30) + g_a \cdot \ln(a/70)]$$

where $k_d = 9.257 \times 10^{-4}$, $g_e = 0.04543$, $g_a = 1.725$.

References

1. World Health Organization (2012) Preliminary dose estimation from the nuclear accident after the 2011 Great East Japan Earthquake and Tsunami.
2. Akahane K, Yonai S, Fukuda S, Miyahara N, Yasuda H, et al. (2013) NIRS external dose estimation system for Fukushima residents after the Fukushima Dai-ichi NPP accident. Sci Rep 3: 1670.
3. Ministry of Education Culture Sports Science and Technology (2011) http://www.mext.go.jp/b_menu/shingi/chousa/gijyutu/017/shiryo/__icsFiles/afieldfile/2011/09/02/1310688_1.pdf. Accessed: 4 Sep. 2014. [in Japanese]
4. Fukushima Prefecture (2011) http://www.pref.fukushima.lg.jp/sec/16025d/kako-monitoring.html. Accessed: 4 Sep. 2014. [in Japanese]
5. Golikov V, Wallstroem E, Woehni T, Tanaka K, Endo S, et al. (2007) Evaluation of conversion coefficients from measurable to risk quantities for external exposure over contaminated soil by use of physical human phantoms. Radiation and Environmental Biophysics 46: 375-382.
6. Takahara S, Iijima M, Shimada K, Kimura M, Homma T (2014) Chapter 18, Probabilistic Assessment of Doses to the Public Living in Areas Contaminated by the Fukushima Daiichi Nuclear Power Plant Accident. In Radiation monitoring and dose estimation of the Fukushima nuclear accident; Takahashi S, editor: Springer Japan.
7. ICRP (1983) Radionuclide transformations - Energy and intensity of emissions. ICRP Publication 38, Ann. ICRP 11-13.
8. Nuclear Emergency Response Headquarters (2011) Prediction of future air dose rate from current air dose. [in Japanese]
9. World Health Organization (2013) Health risk assessment from the nuclear accident after the 2011 Great East Japan Earthquake and Tsunami based on a preliminary

dose estimation.
10. Harada KH, Niisoe T, Imanaka M, Takahashi T, Amako K, et al. (2014) Radiation dose rates now and in the future for residents neighboring restricted areas of the Fukushima Daiichi nuclear power plant. P Natl Acad Sci USA 111: E914-E923.
