# Supplementary material for: Estimated Dietary Intake of Radionuclides and Health Risks for the Citizens of Fukushima City, Tokyo, and Osaka after the 2011 Nuclear Accident
Source: PLoS One. 2014 Nov 12;9(11):e112791. doi: 10.1371/journal.pone.0112791 (PMC4229249; doi:10.1371/journal.pone.0112791)
Supplement: Table S1 — Thyroid equivalent dose coefficients for ingestion of 131I and the effective dose coefficients for ingestion of 131I, 134Cs and 137Cs (µSv/Bq). (PDF) [file pone.0112791.s012.pdf]

Table S1. Thyroid equivalent dose coefficients for ingestion of ^{131}I and the effective dose coefficients for ingestion of ^{131}I, ^{134}Cs and ^{137}Cs (μSv/Bq).

	thyroid equivalent dose coefficients for ingestion of ^{131}I	effective dose coefficients for ingestion of ^{131}I	effective dose coefficients for ingestion of ^{134}Cs	effective dose coefficients for ingestion of ^{137}Cs
< 1 y	3.7	1.8×10^{-1}	2.6×10^{-2}	2.1×10^{-2}
1-6 y (M)	2.1	1.0×10^{-1}	1.3×10^{-2}	9.6×10^{-3}
1-6 y (F)	2.1	1.0×10^{-1}	1.3×10^{-2}	9.6×10^{-3}
7-12 y (M)	1.0	5.2×10^{-2}	1.4×10^{-2}	1.0×10^{-2}
7-12 y (F)	1.0	5.2×10^{-2}	1.4×10^{-2}	1.0×10^{-2}
13-18 y (M)	6.8×10^{-1}	3.4×10^{-2}	1.9×10^{-2}	1.3×10^{-2}
13-18 y (F)	6.8×10^{-1}	3.4×10^{-2}	1.9×10^{-2}	1.3×10^{-2}
≥ 19 y (M)	4.3×10^{-1}	2.2×10^{-2}	1.9×10^{-2}	1.3×10^{-2}
≥ 19 y (F)	4.3×10^{-1}	2.2×10^{-2}	1.9×10^{-2}	1.3×10^{-2}
Pregnant	4.3×10^{-1}	2.2×10^{-2}	1.9×10^{-2}	1.3×10^{-2}

References

ICRP (1993) Age-dependent doses to members of the public from intake of radionuclides: Part 2 ingestion dose coefficients ICRP Publication 67, Ann. ICRP 23(3-4).

ICRP (1996) Age-dependent doses to the members of the public from intake of radionuclides - Part 5 compilation of ingestion and inhalation coefficients, Ann. ICRP 26(1).
