# Supplementary material for: Estimated Dietary Intake of Radionuclides and Health Risks for the Citizens of Fukushima City, Tokyo, and Osaka after the 2011 Nuclear Accident
Source: PLoS One. 2014 Nov 12;9(11):e112791. doi: 10.1371/journal.pone.0112791 (PMC4229249; doi:10.1371/journal.pone.0112791)
Supplement: Table S2 — Daily consumption rates of drinking water and foods, and relative standard deviations (RSDs) of doses received by ingesting the foods in the same areas. (PDF) [file pone.0112791.s013.pdf]

Table S2. Daily consumption rates of drinking water and foods, and relative standard deviations (RSDs) of doses received by ingesting the foods in the same areas.

	Daily consumption rates of drinking water and foods per person (g/d)										RSD of doses
	< 1 y	1-6 y (M)	1-6 y (F)	7-12 y (M)	7-12 y (F)	13-18 y (M)	13-18 y (F)	$\geq$ 19 y (M)[a]	$\geq$ 19 y (F)	Pregnant	
Drinking water	840	1340	1300	2190	2140	2410	2180	2360 ± 1150[b]	2190	2100	-
Grains											
Rice	69	196	168	319	276	499	324	424 ± 209[c]	292	228	112%
Other grains	21	83	82	128	111	128	111	128 ± 142[c]	111	142	75%
Vegetables											
Potato	13	37	34	85	78	79	68	60 ± 78[c]	56	58	445%
Spinach	0.19	2.3	2.1	4.2	4.1	4.7	4.4	4.8 ± 2.9[c]	4.4	4.4	50%
Garland chrysanthemum and ging-geng-cai	0.09	1.1	1.0	2.0	2.0	2.3	2.1	2.3 ± 1.4[c]	2.1	2.1	52%
Mustard spinach and non-heading lettuce	0.25	3.1	2.8	5.6	5.4	6.2	5.7	6.4 ± 3.8[c]	5.8	5.7	140%
Heading leafy vegetables	4.1	49	44	90	87	100	92	102 ± 61[c]	93	92	63%
Broccoli and cauliflower	0.28	3.4	3.1	6.2	6.1	7.0	6.4	7.1 ± 4.2[c]	6.5	6.4	200%
Naganegi onion, chivee and asparagus	0.80	9.6	8.6	17	17	20	18	20 ± 12[c]	18	18	70%
Turnip	0.21	1.8	1.7	3.3	3.2	3.7	3.3	4.0 ± 2.4[c]	3.7	3.2	138%
Bamboo shoots	0.037	0.30	0.29	0.56	0.55	0.63	0.56	0.69 ± 0.41[c]	0.64	0.55	60%
Other root crops	4.2	35	33	65	64	73	65	80 ± 48[c]	74	63	245%
Beans	10.0	29	28	66	63	64	62	64 ± 81[c]	62	48	50%
Kiwifruit	0.57	1.5	1.5	1.3	1.4	1.3	1.3	2.0 ± 2.8[c]	2.1	2.0	84%
Chestnut	0.19	0.50	0.51	0.43	0.46	0.43	0.45	0.66 ± 0.92[c]	0.70	0.66	115%
Other fruit vegetables	66	173	177	150	159	148	154	227 ± 318[c]	240	228	364%
Milk and dairy products											
Milk	5.8	160	139	308	260	216	152	82 ± 124[c]	87	100	167%
Dairy products	22	53	47	28	35	26	36	31 ± 46[c]	39	47	167%
Formula milk	114	0	0	0	0	0	0	0 ± 0	0	0	-
Meat and eggs											
Beef	0.10	10	7.9	16	15	27	19	18 ± 15[c]	12	21	53%
Pork	0.70	37	32	51	43	68	51	47 ± 39[c]	36	44	98%
Chicken	2.0	14	14	24	23	39	31	22 ± 19[c]	16	22	136%
Chicken eggs	2.9	28	24	36	32	51	47	40 ± 38[c]	35	39	42%
Fisheries products											
Wild *ayu*, wild Japanese dace and wild landlocked *masu* salmon	0.34	0.37	0.40	0.60	0.54	0.70	0.63	1.1 ± 1.0[c]	0.87	0.52	88%
Other fresh fisheries products	2.7	2.8	3.1	4.6	4.2	5.4	4.9	8.3 ± 7.6[c]	6.7	4.0	160%
Marine products	9.7	38	40	76	67	82	72	111 ± 101[c]	90	54	218%
Tea[e]	189	266	266	439	439	439	439	439 ± 386[d]	439	439	37%
Mushrooms											
Shiitake mushroom (virgin wood)	0.10	0.19	0.19	0.31	0.31	0.36	0.36	0.47 ± 0.77[c]	0.47	0.47	87%
Other mushrooms	3.0	6.0	6.0	9.6	9.6	11	11	15 ± 24[c]	15	15	206%

a: arithmetic mean ± standard deviation.

b: calculated from RSD in ref[1].

c: calculated from RSD in ref[2].

d: calculated from RSD in ref[3].

e: 10 g of leaf is used to make 300 g of tea and 60% of radionuclides in the leaf enter the tea.

References

1. Murakami M, Takeda H, Okaneya M, Kobayashi Y, Oki T (2012) Classification and motives of drink intakes based on the situations using behavioral record. SEISAM-KENKYU 64: 359-366. [in Japanese]
2. Ministry of Health and Welfare (2012) The national health and nutrition survey in Japan, 2010. [in Japanese]
3. Suzuki K, Okuda T, HIgasine Y (2006) The daily intakes of drinks and folate from the drinks by young women. Mem Osaka Kyoiku Univ Ser2 Soc Sci Home Econ

54: 27-34. [in Japanese]
