# Supplementary material for: Estimated Dietary Intake of Radionuclides and Health Risks for the Citizens of Fukushima City, Tokyo, and Osaka after the 2011 Nuclear Accident
Source: PLoS One. 2014 Nov 12;9(11):e112791. doi: 10.1371/journal.pone.0112791 (PMC4229249; doi:10.1371/journal.pone.0112791)
Supplement: Table S3 — Numbers of samples analyzed each month. (PDF) [file pone.0112791.s014.pdf]

Table S3. Numbers of samples analyzed each month.

	2011										2012		
	Mar.	Apr.	May	Jun.	Jul.	Aug.	Sep.	Oct.	Nov.	Dec.	Jan.	Feb.	Mar.
Rice	0	0	0	0	0	524	1875	976	82	20	4	39	13
Other grains	0	0	0	37	138	104	269	4	3	0	2	0	0
Potato	0	0	0	68	117	38	150	173	147	86	50	97	46
Spinach	103	286	246	193	23	7	19	65	102	81	64	92	52
Garland chrysanthemum and ging-geng-cai	23	41	22	25	3	2	13	38	58	42	31	36	25
Mustard spinach and non-heading lettuce	46	78	47	60	26	17	31	27	47	29	31	68	35
Heading leafy vegetables	46	83	105	83	30	21	34	133	167	103	85	100	50
Broccoli and cauliflower	16	32	50	49	5	3	18	63	85	34	16	18	12
Naganegi onion, chivee and asparagus	59	51	66	59	57	40	91	96	99	69	72	107	46
Turnip	2	21	58	27	12	4	10	106	62	30	15	22	13
Bamboo shoots	0	0	78	49	2	0	2	0	0	0	0	0	3
Other root crops	3	1	18	50	43	44	63	171	162	0	107	261	145
Beans	0	9	38	47	78	79	55	115	418	91	5	24	11
Kiwifruit	0	0	0	0	0	0	2	37	32	8	2	1	0
Chestnut	0	0	0	0	0	2	89	8	6	0	0	0	0
Other fruit vegetables	73	104	110	217	644	755	617	525	504	354	165	170	117
Milk and dairy products	168	107	121	103	124	108	122	135	137	129	149	188	101
Formula milk	0	0	0	0	0	0	0	0	0	6	9	0	0
Beef	5	16	24	12	787	3907	4526	9579	13507	13690	7826	9368	6430
Pork	7	27	20	18	30	61	43	54	39	24	33	64	28
Chicken	8	8	1	15	11	12	17	11	19	19	16	30	16
Chicken eggs	9	22	3	12	14	22	32	21	41	42	37	58	38
Wild *ayu*, wild Japanese dace and wild landlocked *masu* salmon	0	4	38	54	49	27	29	10	10	3	0	43	76
Other fresh fisheries products	2	8	16	33	45	28	51	31	42	36	23	79	62
Marine products	16	171	198	291	286	408	615	927	1157	855	600	1263	573
Tea	0	0	83	135	33	17	116	1520	221	57	0	3	0
Mushrooms	0	36	79	11	50	15	50	62	74	79	21	86	83
Shiitake mushroom (virgin wood)	2	10	11	9	12	64	51	153	42	45	13	50	26
