# Supplementary material for: Estimated Dietary Intake of Radionuclides and Health Risks for the Citizens of Fukushima City, Tokyo, and Osaka after the 2011 Nuclear Accident
Source: PLoS One. 2014 Nov 12;9(11):e112791. doi: 10.1371/journal.pone.0112791 (PMC4229249; doi:10.1371/journal.pone.0112791)
Supplement: Table S4 — Numbers of samples (leafy vegetables, fruit vegetables, milk and dairy products, meat and eggs, and marine products) analyzed in each prefecture in March and April 2011. (PDF) [file pone.0112791.s015.pdf]

Table S4. Numbers of samples (leafy vegetables, fruit vegetables, milk and dairy products, meat and eggs, and marine products) analyzed in each prefecture in March and April 2011.

	Fukushima	Ibaraki	Tochigi	Gunma	Saitama	Chiba	Tokyo	Kanagawa	Other 8 prefectures
Spinach	105	86	39	51	32	36	6	13	21
Garland chrysanthemum and ging-geng-cai	2	6	14	17	1	22	0	0	2
Mustard spinach and non-heading lettuce	42	18	0	2	23	9	13	10	7
Heading leafy vegetables	55	57	3	2	0	8	0	4	0
Broccoli and cauliflower	44	0	0	1	3	0	0	0	0
Naganegi onion, chivee and asparagus	44	15	12	17	16	2	0	0	4
Other fruit vegetables	75	13	20	44	11	5	0	0	9
Milk and dairy products	171	39	9	10	10	7	2	17	10
Beef	15	2	1	1	0	0	0	1	1
Pork	28	2	1	1	0	0	0	2	0
Chicken	14	1	0	1	0	0	0	0	0
Chicken eggs	27	2	1	1	0	0	0	0	0
Marine products	11	111	0	0	0	47	2	14	2
