# Supplementary material for: Estimated Dietary Intake of Radionuclides and Health Risks for the Citizens of Fukushima City, Tokyo, and Osaka after the 2011 Nuclear Accident
Source: PLoS One. 2014 Nov 12;9(11):e112791. doi: 10.1371/journal.pone.0112791 (PMC4229249; doi:10.1371/journal.pone.0112791)
Supplement: Table S5 — Average thyroid equivalent doses of 131I without countermeasures in Fukushima City (Case 1) in the first year after the accident (µSv). M, male; F, female. Case 1, citizens consumed vegetables bought from markets. (PDF) [file pone.0112791.s016.pdf]

Table S5. Average thyroid equivalent doses of ^{131}I without countermeasures in Fukushima City (Case 1) in the first year after the accident (μSv). M, male; F, female. Case 1, citizens consumed vegetables bought from markets.

	< 1 y	1-6 y (M)	1-6 y (F)	7-12 y (M)	7-12 y (F)	13-18 y (M)	13-18 y (F)	≥ 19 y (M)	≥ 19 y (F)	Pregnant
Drinking water	1400	1300	1300	1000	990	760	690	470	430	420
Grain	0	0	0	0	0	0	0	0	0	0
Vegetable[a]	250	1300	1200	1100	1100	840	770	550	510	500
	(60)	(390)	(350)	(340)	(330)	(260)	(240)	(170)	(150)	(150)
Milk and dairy product[a]	190	1200	1000	990	850	480	350	130	140	160
	(80)	(490)	(430)	(400)	(350)	(200)	(150)	(50)	(60)	(70)
Meat and egg	0	10	10	0	0	0	0	0	0	0
Fishery product	0	10	10	10	10	0	0	0	0	0
Tea	0	0	0	0	0	0	0	0	0	0
Mushroom	0	0	0	0	0	0	0	0	0	0
Total[a]	1900	3800	3500	3100	2900	2100	1800	1200	1100	1100
	(130)	(880)	(780)	(750)	(680)	(450)	(380)	(220)	(210)	(220)

a Values in parenthesis represent doses from 17th March 2011 to 20th March 2011.
