# Supplementary material for: Estimated Dietary Intake of Radionuclides and Health Risks for the Citizens of Fukushima City, Tokyo, and Osaka after the 2011 Nuclear Accident
Source: PLoS One. 2014 Nov 12;9(11):e112791. doi: 10.1371/journal.pone.0112791 (PMC4229249; doi:10.1371/journal.pone.0112791)
Supplement: Table S6 — Average effective doses of 134Cs and 137Cs without countermeasures in Fukushima City (Case 1) in the first year after the accident (µSv). M, male; F, female. Case 1, citizens consumed vegetables bought from markets. (PDF) [file pone.0112791.s017.pdf]

Table S6. Average effective doses of $^{134}$Cs and $^{137}$Cs without countermeasures in Fukushima City (Case 1) in the first year after the accident (μSv). M, male; F, female. Case 1, citizens consumed vegetables bought from markets.

| | < 1 y | 1-6 y (M) | 1-6 y (F) | 7-12 y (M) | 7-12 y (F) | 13-18 y (M) | 13-18 y (F) | ≥ 19 y (M) | ≥ 19 y (F) | Pregnant |
|---|---|---|---|---|---|---|---|---|---|---|
| Drinking water | 0.75 | 0.57 | 0.55 | 0.98 | 0.96 | 1.4 | 1.3 | 1.4 | 1.3 | 1.3 |
| Grain | 0.10 | 0.16 | 0.14 | 0.26 | 0.23 | 0.48 | 0.34 | 0.43 | 0.31 | 0.30 |
| Vegetable[a] | 4.2 | 13 | 12 | 21 | 21 | 31 | 29 | 34 | 32 | 31 |
| | (0.16) | (0.91) | (0.82) | (1.76) | (1.72) | (2.62) | (2.41) | (2.68) | (2.44) | (2.41) |
| Milk and dairy product[a] | 0.15 | 0.80 | 0.70 | 1.48 | 1.27 | 1.40 | 1.04 | 0.60 | 0.65 | 0.76 |
| | (0.02) | (0.11) | (0.10) | (0.20) | (0.17) | (0.19) | (0.14) | (0.08) | (0.09) | (0.10) |
| Meat and egg | 0.04 | 1.4 | 1.1 | 2.2 | 2.1 | 4.9 | 3.5 | 3.2 | 2.3 | 3.7 |
| Fishery product | 0.87 | 0.62 | 0.66 | 1.2 | 1.0 | 1.8 | 1.6 | 2.6 | 2.1 | 1.2 |
| Tea | 0.42 | 0.29 | 0.29 | 0.50 | 0.50 | 0.67 | 0.67 | 0.67 | 0.67 | 0.67 |
| Mushroom | 0.13 | 0.13 | 0.13 | 0.22 | 0.22 | 0.34 | 0.34 | 0.45 | 0.45 | 0.45 |
| Total[a] | 6.7 | 17 | 15 | 28 | 27 | 42 | 38 | 43 | 39 | 39 |
| | (0.18) | (1.0) | (0.9) | (2.0) | (1.9) | (2.8) | (2.5) | (2.8) | (2.5) | (2.5) |

a Values in parenthesis represent doses from 17th March 2011 to 20th March 2011.
