# Supplementary material for: Estimated Dietary Intake of Radionuclides and Health Risks for the Citizens of Fukushima City, Tokyo, and Osaka after the 2011 Nuclear Accident
Source: PLoS One. 2014 Nov 12;9(11):e112791. doi: 10.1371/journal.pone.0112791 (PMC4229249; doi:10.1371/journal.pone.0112791)
Supplement: Table S7 — Average thyroid equivalent doses of 131I with countermeasures in Fukushima City (Case 1) in the first year after the accident (µSv). M, male; F, female. Case 1, citizens consumed vegetables bought from markets. (PDF) [file pone.0112791.s018.pdf]

Table S7. Average thyroid equivalent doses of ^{131}I with countermeasures in Fukushima City (Case 1) in the first year after the accident (μSv). M, male; F, female. Case 1, citizens consumed vegetables bought from markets.

	< 1 y	1-6 y (M)	1-6 y (F)	7-12 y (M)	7-12 y (F)	13-18 y (M)	13-18 y (F)	≥ 19 y (M)	≥ 19 y (F)	Pregnant
Drinking water	1400	1300	1300	1000	990	760	690	470	430	420
Grain	0	0	0	0	0	0	0	0	0	0
Vegetable[a]	170	760	700	610	600	460	430	310	290	280
	(60)	(390)	(350)	(340)	(330)	(260)	(240)	(170)	(150)	(150)
Milk and dairy product[a]	90	580	500	480	410	230	170	60	70	80
	(80)	(490)	(430)	(400)	(350)	(200)	(150)	(50)	(60)	(70)
Meat and egg	0	10	10	0	0	0	0	0	0	0
Fishery product	0	10	10	10	10	0	0	0	0	0
Tea	0	0	0	0	0	0	0	0	0	0
Mushroom	0	0	0	0	0	0	0	0	0	0
Total[a]	1700	2700	2500	2100	2000	1500	1300	840	790	780
	(130)	(880)	(780)	(750)	(680)	(450)	(380)	(220)	(210)	(220)

a Values in parenthesis represent doses from 17th March 2011 to 20th March 2011.
