# Supplementary material for: Estimated Dietary Intake of Radionuclides and Health Risks for the Citizens of Fukushima City, Tokyo, and Osaka after the 2011 Nuclear Accident
Source: PLoS One. 2014 Nov 12;9(11):e112791. doi: 10.1371/journal.pone.0112791 (PMC4229249; doi:10.1371/journal.pone.0112791)
Supplement: Table S8 — Average effective doses of 134Cs and 137Cs with countermeasures in Fukushima City (Case 1) in the first year after the accident (µSv). M, male; F, female. Case 1, citizens consumed vegetables bought from markets. (PDF) [file pone.0112791.s019.pdf]

Table S8. Average effective doses of $^{134}Cs$ and $^{137}Cs$ with countermeasures in Fukushima City (Case 1) in the first year after the accident (μSv). M, male; F, female. Case 1, citizens consumed vegetables bought from markets.

| | < 1 y | 1-6 y (M) | 1-6 y (F) | 7-12 y (M) | 7-12 y (F) | 13-18 y (M) | 13-18 y (F) | ≥ 19 y (M) | ≥ 19 y (F) | Pregnant |
|---|---|---|---|---|---|---|---|---|---|---|
| Drinking water | 0.75 | 0.57 | 0.55 | 0.98 | 0.96 | 1.4 | 1.3 | 1.4 | 1.3 | 1.3 |
| Grain | 0.05 | 0.08 | 0.08 | 0.13 | 0.12 | 0.21 | 0.16 | 0.20 | 0.16 | 0.17 |
| Vegetable[a] | 2.9 | 5.1 | 5.0 | 6.7 | 6.8 | 9.4 | 9.1 | 12 | 11 | 11 |
| | (0.16) | (0.91) | (0.82) | (1.8) | (1.7) | (2.6) | (2.4) | (2.7) | (2.4) | (2.4) |
| Milk and dairy product[a] | 0.10 | 0.53 | 0.47 | 0.98 | 0.85 | 0.93 | 0.69 | 0.40 | 0.43 | 0.50 |
| | (0.02) | (0.11) | (0.10) | (0.20) | (0.17) | (0.19) | (0.14) | (0.08) | (0.09) | (0.10) |
| Meat and egg | 0.03 | 1.1 | 0.86 | 1.7 | 1.5 | 3.5 | 2.5 | 2.3 | 1.7 | 2.6 |
| Fishery product | 0.86 | 0.62 | 0.66 | 1.2 | 1.0 | 1.7 | 1.6 | 2.5 | 2.1 | 1.2 |
| Tea | 0.37 | 0.25 | 0.25 | 0.44 | 0.44 | 0.58 | 0.58 | 0.58 | 0.58 | 0.58 |
| Mushroom | 0.04 | 0.04 | 0.04 | 0.07 | 0.07 | 0.11 | 0.11 | 0.15 | 0.15 | 0.15 |
| Total[a] | 5.1 | 8.2 | 7.9 | 12 | 12 | 18 | 16 | 19 | 18 | 17 |
| | (0.18) | (1.0) | (0.91) | (2.0) | (1.9) | (2.8) | (2.5) | (2.8) | (2.5) | (2.5) |

a Values in parenthesis represent doses from 17th March 2011 to 20th March 2011.
