# Supplementary material for: Estimated Dietary Intake of Radionuclides and Health Risks for the Citizens of Fukushima City, Tokyo, and Osaka after the 2011 Nuclear Accident
Source: PLoS One. 2014 Nov 12;9(11):e112791. doi: 10.1371/journal.pone.0112791 (PMC4229249; doi:10.1371/journal.pone.0112791)
Supplement: Table S10 — Average effective doses of 134Cs and 137Cs without countermeasures in Fukushima City (Case 2) in the first year after the accident (µSv). M, male; F, female. Case 2, citizens consumed vegetables grown locally. (PDF) [file pone.0112791.s021.pdf]

Table S10. Average effective doses of ^{134}Cs and ^{137}Cs without countermeasures in Fukushima City (Case 2) in the first year after the accident (μSv). M, male; F, female. Case 2, citizens consumed vegetables grown locally.

	< 1 y	1-6 y (M)	1-6 y (F)	7-12 y (M)	7-12 y (F)	13-18 y (M)	13-18 y (F)	≥ 19 y (M)	≥ 19 y (F)	Pregnant
Drinking water	0.75	0.57	0.55	0.98	0.96	1.4	1.3	1.4	1.3	1.3
Grain	0.10	0.16	0.14	0.26	0.23	0.48	0.34	0.43	0.31	0.30
Vegetable[a]	13	42	39	73	72	110	100	120	110	100
	(0.73)	(4.2)	(3.8)	(8.2)	(8.0)	(12)	(11)	(12)	(11)	(11)
Milk and dairy product[a]	0.15	0.80	0.70	1.48	1.27	1.40	1.04	0.60	0.65	0.76
	(0.02)	(0.11)	(0.10)	(0.20)	(0.17)	(0.19)	(0.14)	(0.08)	(0.09)	(0.10)
Meat and egg	0.04	1.4	1.1	2.2	2.1	4.9	3.5	3.2	2.3	3.7
Fishery product	0.87	0.62	0.66	1.2	1.0	1.8	1.6	2.6	2.1	1.2
Tea	0.42	0.29	0.29	0.50	0.50	0.67	0.67	0.67	0.67	0.67
Mushroom	0.13	0.13	0.13	0.22	0.22	0.34	0.34	0.45	0.45	0.45
Total[a]	16	46	43	80	79	120	110	130	120	110
	(0.75)	(4.3)	(3.9)	(8.4)	(8.1)	(12)	(11)	(12)	(11)	(11)

a Values in parenthesis represent doses from 17th March 2011 to 20th March 2011.
