# Supplementary material for: Estimated Dietary Intake of Radionuclides and Health Risks for the Citizens of Fukushima City, Tokyo, and Osaka after the 2011 Nuclear Accident
Source: PLoS One. 2014 Nov 12;9(11):e112791. doi: 10.1371/journal.pone.0112791 (PMC4229249; doi:10.1371/journal.pone.0112791)
Supplement: Table S11 — Average thyroid equivalent doses of 131I with countermeasures in Fukushima City (Case 2) in the first year after the accident (µSv). M, male; F, female. Case 2, citizens consumed vegetables grown locally. (PDF) [file pone.0112791.s022.pdf]

Table S11. Average thyroid equivalent doses of ^{131}I with countermeasures in Fukushima City (Case 2) in the first year after the accident (μSv). M, male; F, female. Case 2, citizens consumed vegetables grown locally.

	< 1 y	1-6 y (M)	1-6 y (F)	7-12 y (M)	7-12 y (F)	13-18 y (M)	13-18 y (F)	≥ 19 y (M)	≥ 19 y (F)	Pregnant
Drinking water	1400	1300	1300	1000	990	760	690	470	430	420
Grain	0	0	0	0	0	0	0	0	0	0
Vegetable[a]	920	5300	4800	4400	4300	3400	3100	2200	2000	2000
	(280)	(1900)	(1700)	(1700)	(1600)	(1300)	(1200)	(820)	(740)	(730)
Milk and dairy product[a]	90	580	500	480	410	230	170	60	70	80
	(80)	(490)	(430)	(400)	(350)	(200)	(150)	(50)	(60)	(70)
Meat and egg	0	10	10	0	0	0	0	0	0	0
Fishery product	0	10	10	10	10	0	0	0	0	0
Tea	0	0	0	0	0	0	0	0	0	0
Mushroom	0	0	0	0	0	0	0	0	0	0
Total[a]	2500	7200	6500	5900	5700	4400	4000	2700	2500	2500
	(360)	(2400)	(2200)	(2100)	(2000)	(1500)	(1300)	(870)	(800)	(800)

a Values in parenthesis represent doses from 17th March 2011 to 20th March 2011.
