# Supplementary material for: Estimated Dietary Intake of Radionuclides and Health Risks for the Citizens of Fukushima City, Tokyo, and Osaka after the 2011 Nuclear Accident
Source: PLoS One. 2014 Nov 12;9(11):e112791. doi: 10.1371/journal.pone.0112791 (PMC4229249; doi:10.1371/journal.pone.0112791)
Supplement: Table S13 — Average thyroid equivalent doses of 131I without countermeasures in Tokyo in the first year after the accident (µSv). M, male; F, female. (PDF) [file pone.0112791.s024.pdf]

Table S13. Average thyroid equivalent doses of $^{131}I$ without countermeasures in Tokyo in the first year after the accident (μSv). M, male; F, female.

| | < 1 y | 1-6 y (M) | 1-6 y (F) | 7-12 y (M) | 7-12 y (F) | 13-18 y (M) | 13-18 y (F) | ≥ 19 y (M) | ≥ 19 y (F) | Pregnant |
|---|---|---|---|---|---|---|---|---|---|---|
| Drinking water | 690 | 620 | 610 | 490 | 470 | 360 | 330 | 220 | 210 | 200 |
| Grain | 0 | 0 | 0 | 0 | 0 | 0 | 0 | 0 | 0 | 0 |
| Vegetable[a] | 80 | 410 | 370 | 330 | 330 | 250 | 230 | 170 | 150 | 150 |
| | (10) | (100) | (90) | (80) | (80) | (60) | (60) | (40) | (40) | (40) |
| Milk and dairy product[a] | 40 | 250 | 220 | 210 | 180 | 100 | 70 | 30 | 30 | 30 |
| | (10) | (80) | (70) | (60) | (50) | (30) | (20) | (10) | (10) | (10) |
| Meat and egg | 0 | 0 | 0 | 0 | 0 | 0 | 0 | 0 | 0 | 0 |
| Fishery product | 0 | 10 | 10 | 10 | 10 | 0 | 0 | 0 | 0 | 0 |
| Tea | 0 | 0 | 0 | 0 | 0 | 0 | 0 | 0 | 0 | 0 |
| Mushroom | 0 | 0 | 0 | 0 | 0 | 0 | 0 | 0 | 0 | 0 |
| Total[a] | 810 | 1280 | 1200 | 1030 | 980 | 720 | 640 | 420 | 390 | 390 |
| | (30) | (170) | (150) | (150) | (140) | (90) | (80) | (50) | (50) | (50) |

a Values in parenthesis represent doses from 18th March 2011 to 20th March 2011.
