# Supplementary material for: Estimated Dietary Intake of Radionuclides and Health Risks for the Citizens of Fukushima City, Tokyo, and Osaka after the 2011 Nuclear Accident
Source: PLoS One. 2014 Nov 12;9(11):e112791. doi: 10.1371/journal.pone.0112791 (PMC4229249; doi:10.1371/journal.pone.0112791)
Supplement: Table S15 — Average thyroid equivalent doses of 131I with countermeasures in Tokyo in the first year after the accident (µSv). M, male; F, female. (PDF) [file pone.0112791.s026.pdf]

Table S15. Average thyroid equivalent doses of ^{131}I with countermeasures in Tokyo in the first year after the accident (μSv). M, male; F, female.

	< 1 y	1-6 y (M)	1-6 y (F)	7-12 y (M)	7-12 y (F)	13-18 y (M)	13-18 y (F)	≥ 19 y (M)	≥ 19 y (F)	Pregnant
Drinking water	510	620	610	490	470	360	330	220	210	200
Grain	0	0	0	0	0	0	0	0	0	0
Vegetable[a]	70	310	290	250	250	190	180	130	120	120
	(10)	(100)	(90)	(80)	(80)	(60)	(60)	(40)	(40)	(40)
Milk and dairy product[a]	30	160	140	140	120	70	50	20	20	20
	(10)	(80)	(70)	(60)	(50)	(30)	(20)	(10)	(10)	(10)
Meat and egg	0	0	0	0	0	0	0	0	0	0
Fishery product	0	10	10	10	10	0	0	0	0	0
Tea	0	0	0	0	0	0	0	0	0	0
Mushroom	0	0	0	0	0	0	0	0	0	0
Total[a]	600	1110	1040	880	840	620	560	370	350	340
	(30)	(170)	(150)	(150)	(140)	(90)	(80)	(50)	(50)	(50)

a Values in parenthesis represent doses from 18th March 2011 to 20th March 2011.
