# Supplementary material for: Estimated Dietary Intake of Radionuclides and Health Risks for the Citizens of Fukushima City, Tokyo, and Osaka after the 2011 Nuclear Accident
Source: PLoS One. 2014 Nov 12;9(11):e112791. doi: 10.1371/journal.pone.0112791 (PMC4229249; doi:10.1371/journal.pone.0112791)
Supplement: Table S17 — Average thyroid equivalent doses of 131I without countermeasures in Osaka in the first year after the accident (µSv). M, male; F, female. (PDF) [file pone.0112791.s028.pdf]

Table S17. Average thyroid equivalent doses of ^{131}I without countermeasures in Osaka in the first year after the accident (μSv). M, male; F, female.

	< 1 y	1-6 y (M)	1-6 y (F)	7-12 y (M)	7-12 y (F)	13-18 y (M)	13-18 y (F)	≥ 19 y (M)	≥ 19 y (F)	Pregnant
Drinking water	0	0	0	0	0	0	0	0	0	0
Grain	0	0	0	0	0	0	0	0	0	0
Vegetable[a]	13	58	53	46	45	35	32	23	22	21
	(3)	(18)	(17)	(16)	(16)	(12)	(11)	(8)	(7)	(7)
Milk and dairy product[a]	0	2	2	2	1	1	1	0	0	0
	(0)	(1)	(1)	(1)	(0)	(0)	(0)	(0)	(0)	(0)
Meat and egg	0	0	0	0	0	0	0	0	0	0
Fishery product	1	2	2	2	2	1	1	1	1	1
Tea	0	0	0	0	0	0	0	0	0	0
Mushroom	0	0	0	0	0	0	0	0	0	0
Total[a]	14	62	57	50	49	37	34	25	23	22
	(3)	(19)	(17)	(16)	(16)	(12)	(11)	(8)	(7)	(7)

a Values in parenthesis represent doses from 18th March 2011 to 20th March 2011.
