# Supplementary material for: Estimated Dietary Intake of Radionuclides and Health Risks for the Citizens of Fukushima City, Tokyo, and Osaka after the 2011 Nuclear Accident
Source: PLoS One. 2014 Nov 12;9(11):e112791. doi: 10.1371/journal.pone.0112791 (PMC4229249; doi:10.1371/journal.pone.0112791)
Supplement: Table S20 — Average effective doses of 134Cs and 137Cs with countermeasures in Osaka in the first year after the accident (µSv). M, male; F, female. (PDF) [file pone.0112791.s031.pdf]

Table S20. Average effective doses of ^{134}Cs and ^{137}Cs with countermeasures in Osaka in the first year after the accident (μSv). M, male; F, female.

	< 1 y	1-6 y (M)	1-6 y (F)	7-12 y (M)	7-12 y (F)	13-18 y (M)	13-18 y (F)	≥ 19 y (M)	≥ 19 y (F)	Pregnant
Drinking water	0.00	0.00	0.00	0.00	0.00	0.00	0.00	0.00	0.00	0.00
Grain	0.05	0.08	0.08	0.13	0.12	0.21	0.16	0.20	0.16	0.17
Vegetable[a]	0.20	0.29	0.29	0.34	0.35	0.46	0.46	0.60	0.61	0.58
	(0.00)	(0.01)	(0.00)	(0.01)	(0.01)	(0.02)	(0.01)	(0.02)	(0.01)	(0.01)
Milk and dairy product[a]	0.00	0.01	0.00	0.01	0.01	0.01	0.01	0.00	0.00	0.00
	(0.00)	(0.00)	(0.00)	(0.00)	(0.00)	(0.00)	(0.00)	(0.00)	(0.00)	(0.00)
Meat and egg	0.00	0.05	0.04	0.08	0.08	0.19	0.13	0.12	0.08	0.14
Fishery product	0.05	0.09	0.09	0.19	0.17	0.27	0.24	0.37	0.30	0.18
Tea	0.37	0.25	0.25	0.44	0.44	0.58	0.58	0.58	0.58	0.58
Mushroom	0.00	0.00	0.00	0.00	0.00	0.00	0.00	0.00	0.00	0.00
Total[a]	0.67	0.76	0.75	1.2	1.2	1.7	1.6	1.9	1.7	1.7
	(0.00)	(0.01)	(0.00)	(0.01)	(0.01)	(0.02)	(0.01)	(0.02)	(0.01)	(0.01)

a Values in parenthesis represent doses from 18th March 2011 to 20th March 2011.
