# Supplementary material for: Estimated Dietary Intake of Radionuclides and Health Risks for the Citizens of Fukushima City, Tokyo, and Osaka after the 2011 Nuclear Accident
Source: PLoS One. 2014 Nov 12;9(11):e112791. doi: 10.1371/journal.pone.0112791 (PMC4229249; doi:10.1371/journal.pone.0112791)
Supplement: Table S21 — Reductions of doses by countermeasures (%). M, male; F, female. Case 1, citizens consumed vegetables bought from markets. (PDF) [file pone.0112791.s032.pdf]

Table S21. Reductions of doses by countermeasures (%). M, male; F, female. Case 1, citizens consumed vegetables bought from markets.

	< 1 y	1-6 y (M)	1-6 y (F)	7-12 y (M)	7-12 y (F)	13-18 y (M)	13-18 y (F)	≥ 19 y (M)	≥ 19 y (F)	Pregnant
^{131}I										
Fukushima City (Case 1)	10	31	30	32	32	30	29	27	27	28
Tokyo	26 (22)	14	13	15	14	13	13	11	12	12
Osaka	22	34	33	36	36	37	36	35	35	36
^{134}Cs and ^{137}Cs										
Fukushima City (Case 1)	24	50	49	57	57	57	57	56	55	55
Tokyo	13 (4)	20	18	21	21	25	23	21	19	23
Osaka	15	18	16	19	19	24	20	19	17	18
Total (effective dose)										
Fukushima City (Case 1)	11	33	31	36	35	38	37	39	39	39
Tokyo	25 (21)	14	13	15	15	15	14	14	14	15
Osaka	18	30	29	30	30	30	28	25	23	24

*: Values in parentheses represent effects of the distribution of bottled water for infants. e.g. For total effective dose, 21% represents the effects of the distribution of bottled water and the remaining 4% represents the restriction of food distribution and voluntary withholding of rice.
