# Supplementary material for: Estimated Dietary Intake of Radionuclides and Health Risks for the Citizens of Fukushima City, Tokyo, and Osaka after the 2011 Nuclear Accident
Source: PLoS One. 2014 Nov 12;9(11):e112791. doi: 10.1371/journal.pone.0112791 (PMC4229249; doi:10.1371/journal.pone.0112791)
Supplement: Table S22 — Effective doses in the first year and over the total lifetime (up to 89 y) due to three pathways (µSv). Ages are those in the first year. M, male; F, female. Case 1, citizens consumed vegetables bought from markets. Case 2, citizens consumed vegetables grown locally. (PDF) [file pone.0112791.s033.pdf]

Table S22. Effective doses in the first year and over the total lifetime (up to 89 y) due to three pathways (μSv). Ages are those in the first year. M, male; F, female. Case 1, citizens consumed vegetables bought from markets. Case 2, citizens consumed vegetables grown locally.

			The 1st year	Total lifetime (up to 89 y)
<1 y				
Ingestion	Fukushima City (Case 1)	M	88	170
		F	88	160
	Fukushima City (Case 2)	M	140	630
		F	140	600
Inhalation			250	250
External exposure			6700	29000
Total	Fukushima City (Case 1)	M	7100	30000
		F	7100	30000
	Fukushima City (Case 2)	M	7100	30000
		F	7100	30000
10 y				
Ingestion	Fukushima City (Case 1)	M	120	210
		F	120	200
	Fukushima City (Case 2)	M	390	940
		F	380	890
Inhalation			240	240
External exposure			6000	27000
Total	Fukushima City (Case 1)	M	6400	27000
		F	6400	27000
	Fukushima City (Case 2)	M	6600	28000
		F	6600	28000
20 y				
Ingestion	Fukushima City (Case 1)	M	62	160
		F	58	140
	Fukushima City (Case 2)	M	260	820
		F	240	760
Inhalation			180	180
External exposure			5300	25000
Total	Fukushima City (Case 1)	M	5600	25000
		F	5600	25000
	Fukushima City (Case 2)	M	5800	26000
		F	5800	26000
